# Synthesis, SAR, and Docking Studies Disclose 2-Arylfuran-1,4-naphthoquinones as* In Vitro *Antiplasmodial Hits

**DOI:** 10.1155/2017/7496934

**Published:** 2017-10-31

**Authors:** Tatiane Freitas Borgati, Maria Fernanda Alves do Nascimento, Juma Fortunato Bernardino, Lunamaura Claudia Oliveira Martins, Alex Gutterres Taranto, Alaíde Braga de Oliveira

**Affiliations:** ^1^Departamento de Produtos Farmacêuticos, Faculdade de Farmácia, UFMG, Campus Pampulha, Av. Antônio Carlos 6627, 31270-901 Belo Horizonte, MG, Brazil; ^2^Faculdade de Farmácia, UFSJ, Campus Divinópolis, Rua Sebastião Gonçalves Coelho 400, Chanadour, 35501-296 Divinópolis, MG, Brazil

## Abstract

A total of 28 lapachol-related naphthoquinones with four different scaffolds were synthesized and spectroscopically characterized.* In vitro* antiplasmodial activity was assayed against the chloroquine-resistant* Plasmodium falciparum* W2 strain by the parasite lactate dehydrogenase (*p*LDH) method. Cytotoxicity against Hep G2A16 cell was determined by the MTT assay. All compounds disclosed higher* in vitro* antiplasmodial activity than lapachol.* Ortho-* and* para*-naphthoquinones with a furan ring fused to the quinonoid moiety were more potent than 2-hydroxy-3-(1′-alkenyl)-1,4-naphthoquinones, while* ortho*-furanonaphthoquinones were more cytotoxic. Molecular docking to* Plasmodium* targets* Pf*cyt* bc1* complex and* Pf*DHOD enzyme showed that five out of the 28 naphthoquinones disclosed favorable binding energies. Furanonaphthoquinones endowed with an aryl moiety linked to the furan ring are highlighted as new* in vitro *antiplasmodial lead compounds and warrant further investigation.

## 1. Introduction

**Figure 1:**
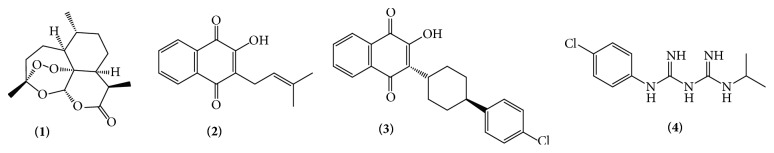
Chemical structures of artemisinin (**1**), lapachol (**2**), atovaquone (**3**), and proguanil (**4**).

Malaria, a tropical disease caused by protozoa* Plasmodium *spp., is still a serious public health problem, affecting mostly Africa (88%) [1].* P. falciparum *is the main agent responsible for mortality and morbidity in endemic tropical regions. Drug-resistance to available agents, including artemisinin (**1**, Figure 1), is a serious limitation to chemotherapeutics [2], the main strategy for combating malaria. Aiming to control selection of artemisinin resistant parasites, ACT (Artemisinin-Based Combination Therapy) was developed and is the first-line treatment recommended by WHO [3].

Lapachol (**2**, Figure 1), a prenylnaphthoquinone occurring in Central and South American species of* Handroanthus* spp. (synon.* Tabebuia,* Bignoniaceae family) [4] and first isolated from* Tabebuia avellanedae,* in 1882 by Paternó [5], was used as an antiplasmodial drug during World War II, when there was a shortage of quinine, the only available antimalarial agent at that time [6]. Since the pioneering work of Wendel (1946), who demonstrated that lapachol (**2**, Figure 1) was active against* P. vivax *[7], numerous publications reported on the antiplasmodial activity of naphthoquinones leading to the development of atovaquone (**3**, Figure 1), a totally synthetic hydroxy-1,4-naphthoquinone [4]. Atovaquone, in combination with proguanil (**4**, Figure 1) (Malarone®), is a recommended regimen for prophylaxis in areas of chloroquine resistance and for treatment of uncomplicated falciparum malaria [8].

Previous studies demonstrated that atovaquone (**3**, Figure 1) is a potent and specific inhibitor of the mitochondrial electron transport at the level of cytochrome* bc*1 complex (*Pf*cyt* bc1*) [9–12]. This inhibition results in the collapse of the mitochondrial membrane potential that causes shutdown of mitochondrial metabolism and blockage of the dihydroorotate dehydrogenase (*Pf*DHOD) enzyme, which participates in the fourth step of the* de novo* pyrimidine biosynthesis, which is essential to parasite nucleic acids synthesis, resulting in parasite death [12, 13].

Atovaquone has a broad spectrum of activity against various apicomplexan parasites and mutations in the* Pf*cyt* bc1* gene were shown to be associated with resistance to atovaquone in various organisms, such as TM90C2B* P. falciparum* strain,* Toxoplasma*, and* Pneumocystis jirovecii *[12–15]. Therefore, atovaquone resistant* P. falciparum* strains represent limitations to its clinical use in malaria endemic areas and are a motivation for the synthesis of atovaquone analogs [16, 17].

In addition to lapachol (**2**, Figure 1), different structural types of naphthoquinones, such as pyran and furanonaphthoquinones, are found in Bignoniaceae [18, 19] and disclose a broad spectrum of biological activities, including antitumor [20–23], trypanocidal [24], anti-inflammatory [7], antifungal [25], and antiparasitic activities [26–33]. Prior studies have reported that furanonaphthoquinones (FNQs) containing alkyl groups linked to the furan ring have proven to be efficient in* in vitro* antiplasmodial evaluations [27, 28, 34]. Furthermore, it has been demonstrated that the incorporation of a basic amino group into naphthoquinones led to new chemical entities with increased* in vitro* antiplasmodial activity, including lapachol derivatives [35–37].

**Figure 2:**
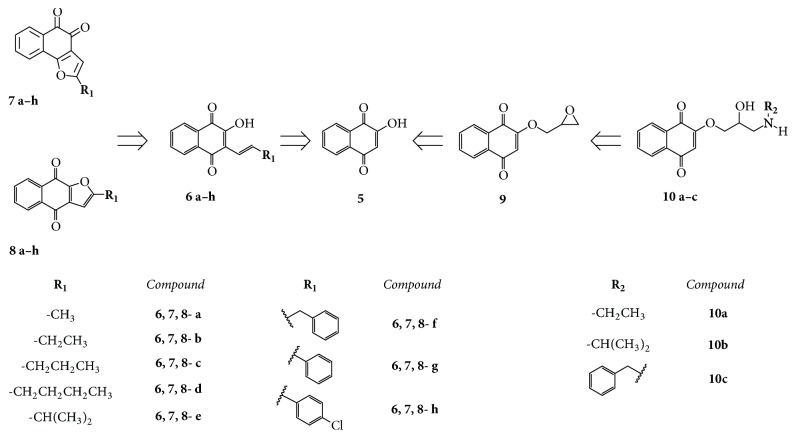
Retrosynthetic scheme of HNQs** 6a–h**,* ortho-*FNQ*s ***7a–h** and* para*-FNQs** 8a–h**, 2-(oxiran-2-ylmethoxy)naphthalene-1,4-dione (**9**), and ANQs** 10a–c,** starting from lawsone (**5**).

The present paper reports the results of an work aiming to investigate the* in vitro* antiplasmodial activity against the resistant strain of* P. falciparum* W2 and the cytotoxicity to Hep G2A16 cell cultures of naphthoquinones with different scaffolds, including* ortho*- and* para*-derivatives represented by* ortho*-FNQs (**7**)* para-*FNQs (**8**) supporting alkyl and aryl groups at the furan ring that were synthesized from HNQs (**6**) obtained from lawsone (**5**), as shown in the retrosynthetic scheme (Figure 2). Nine aryl derivatives (**6**,** 7**,** 8f–h**) were designed as atovaquone analogs. Another group of naphthoquinones, the aminopropoxynaphthoquinones (ANQs)** 12a–c**, derived from** 5** (Figure 2), was synthesized aiming to evaluate the influence of the amino group in the antiplasmodial activity of simple naphthoquinones.

The proposed synthetic work was carried out leading to 28 naphthoquinones of four different scaffolds that were assayed for* in vitro* antiplasmodial activity against chloroquine-resistant* P. falciparum* (W2) and for cytotoxicity to Hep G2A16 cells. Molecular docking simulations for interaction of the synthesized compounds were performed aiming to evaluate the correlation of binding energies with structure activity relationship (SAR) data. Besides* P. falciparum *cytochrome* bc1 *complex (*Pf*cyt* bc1*) that has been proven to be the target for atovaquone [9, 11, 12], docking simulations were also performed against* P. falciparum* dihydroorotate dehydrogenase enzyme (*Pf*DHOD) that is considered an attractive antimalarial chemotherapeutic target [16].

## 2. Materials and Methods

Melting points were determined using a MQAPF-307 melting point apparatus (Microquimica, Brazil) and remained uncorrected. The progress of the reactions was monitored by thin layer chromatography (TLC) in silica gel plates. Column chromatography was performed over silica gel (60–230 mesh). Infrared spectra were recorded on a Spectra One Perkin-Elmer spectrophotometer, fitted with a Paragon ATR accessory. Mass spectra (HRMS) were recorded on a Shimadzu GC MS-QP5050A instrument using direct insertion together with the electrospray ionization method and a quadrupole analyzer. The ^1^H, ^13^C, and DEPT 135 nuclear magnetic resonance (NMR) spectra and two dimensional NMR spectra—correlations spectroscopy (COSY), heteronuclear single-quantum correlation (HSQC), and heteronuclear multiple bound correlation (HMBC)—were recorded on a Bruker Avance DPX 200 and a DPX 400 spectrometer at 200 and 400 MHz using CDCl_3_ and DMSOd_6_ as the solvent and Tetramethylsylane (TMS) as the internal standard, unless otherwise stated. The NMR data are presented as follows: chemical shift in ppm, multiplicity, number of protons, *J* in Hz, and proton assignments. Multiplicities are indicated by the following abbreviations: s (singlet), d (doublet), dd (double doublet), t (triplet), q (quartet), m (multiplet), qn (quintet), st (sextet), ht (heptet), and bs (broad singlet).

### 2.1. General Procedures of Synthesis

#### 2.1.1. 2-Hydroxy-3-(1′-alkenyl)-1,4-naphthoquinones: HNQs (**6a–h**)

**Scheme 1:**
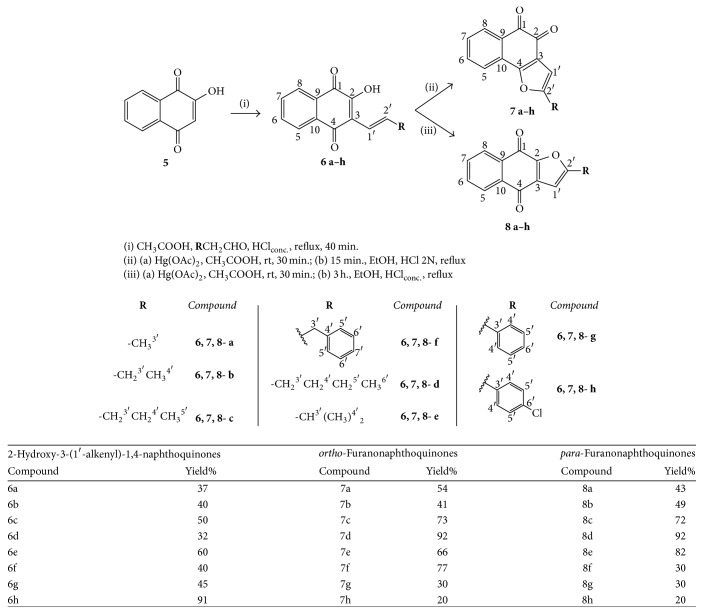
Synthesis of naphthoquinones** 6a–h**,** 7a–h**, and** 8a–h **and their yields.

The appropriated aldehyde (86 mmol) was added to a solution of lawsone (17 mmol) in glacial acetic acid (20 mL), followed by concentrated HCL (15 mL). The reaction mixture was kept under magnetic stirring and reflux for 40 min, at which time the TLC showed the consumption of lawsone. Subsequently, water was added to the reaction mixture (50 mL), followed by extraction with dichloromethane. The organic layer was extracted with aqueous sodium carbonate (2.5%), and the water layers were combined, neutralized with HCl 2N, and extracted with dichloromethane. The organic layer was dried with anhydrous Na_2_SO_4_ and then filtered. The solvent was removed under lower pressure, and the residue was fractionated in a silica gel column chromatography. Fractions eluted with a mixture of hexane/dichloromethane (7 : 3) afforded 2-hydroxy-3-(1′-alkenyl)-1,4-naphthoquinones (**6a–h**, Scheme 1).

#### 2.1.2. Furanonaphthoquinones: FNQs (**7a–h **and** 8a–h**)

Hg(OAc)_2_ (4 mmol) in glacial acetic acid (15 mL) was added to a solution of HNQ (2 mmol) in glacial acetic acid (15 mL). The reaction mixture was kept under magnetic stirring at room temperature for 30 min and at 80°C for 15 min. Subsequently, diethyl ether was added to the reaction mixture. The solid was separated by filtration, and the solution solvent was removed, resulting in a crude product. To obtain the* ortho-*FNQs** 7a–h**, the crude product was dissolved in ethanol (20 mL), and HCl 2N (20 mL) was added. The mixture was kept under magnetic stirring and reflux for 15 min. Subsequently, water (30 mL) was added to the reaction mixture, followed by extraction with ethyl acetate. The organic layer was extracted with aqueous sodium bicarbonate, rinsed with water, and dried with anhydrous Na_2_SO_4_. The solvent was removed, and the residue was chromatographed through a silica gel column. Elution with a mixture of hexane/dichloromethane (8 : 2) led to the* ortho-*furanonaphthoquinones (**7a–h,**Scheme 1). To obtain the* para-*FNQs** 8a–h,** the crude product was dissolved in ethanol (20 mL), and concentrated HCl (20 mL) was added in this solution. The mixture was kept under magnetic stirring and reflux for 3 h. Water (30 mL) was then added to the reaction mixture, followed by extraction with ethyl acetate. The organic layer was extracted using an aqueous sodium bicarbonate, rinsed with water, and dried with anhydrous Na_2_SO_4_. The solvent was removed, and the residue was fractionated in a silica gel column chromatography. Elution with a mixture of hexane/dichloromethane (9 : 1) led to the* para-*FNQs** 8a–h (**Scheme 1).

#### 2.1.3. 2-(Oxiran-2-ylmethoxy)naphthalene-1,4-dione (**9**)

**Scheme 2:**
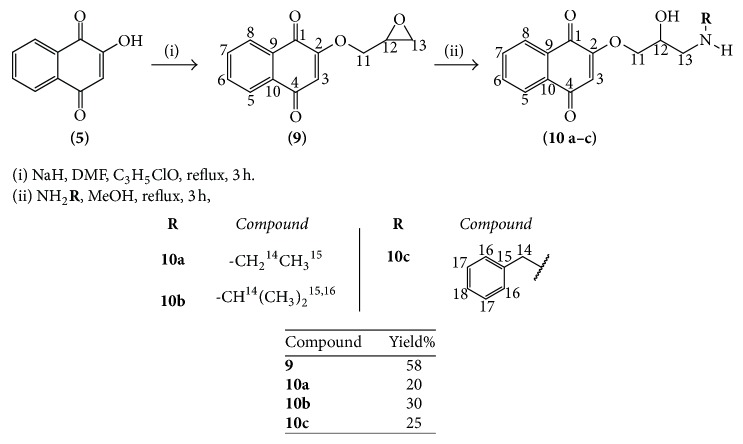
Synthesis of naphthoquinones** 9 **and** 10a–c **and their yields.

Lawsone (6 mmol) was added to a round bottom flask containing sodium hydride (11 mmol) and dimethylformamide (5 mL). The reaction mixture was kept under stirring at room temperature for 30 min, after which time epichlorohydrin (30 mmol) was added to the mixture, which was kept under stirring and reflux for 3 h, when TLC showed the consumption of lawsone. Ethyl acetate (50 mL) was added to the reaction mixture, followed by the extraction with a solution of NaCl. The organic layer was dried with anhydrous Na_2_SO_4_. The solvent was removed, and the residue was purified by silica gel column chromatography. The product was eluted with a mixture of hexane/ethyl acetate (9 : 1), leading to compound** 9 **(Scheme 2).

#### 2.1.4. Aminopropoxynaphthoquinones: ANQS (**10a–c**)

An appropriated amine (0.2 mol) was added to a round bottom flask containing compound** 9** (4 mmol) and MeOH. The reaction solution was kept under stirring and reflux for 3 h, when TLC showed the consumption of starting materials. Ethyl acetate (20 mL) was then added to the mixture, followed by extraction with HCl 2N. The water layer was neutralized with NaOH 2N and extracted with ethyl acetate. The organic layer was dried with anhydrous Na_2_SO_4_. The solvent was removed and the residue was chromatographed through a silica gel column chromatography. The product was eluted with a mixture of hexane/ethyl acetate (9.5 : 0.5), leading to the ANQs** 10a–c** (Scheme 2).

### 2.2. Biological Assays

#### 2.2.1. *In Vitro* Antiplasmodial Activity

There are several* in vitro *methods commonly used for the assessment of parasite growth inhibition [38]. The parasite protein lactate dehydrogenase (*p*LDH) method [39, 40] was chosen to evaluate the* in vitro* antiplasmodial activity of HNQs** 6a–h**,* ortho-*FNQs** 7a–h**,** 8a–h**,** 9**, and ANQs** 10a–c.**

*P. falciparum* (W2 strain) was maintained in a continuous culture, as described by Trager and Jensen (1976) [41]. Ring-stage parasites in sorbitol-synchronized blood cultures were added to 96-well culture plates, at 2% parasitemia and 1% hematocrit. Stock solutions at 50 mg·mL^−1^ of each compound in DMSO were diluted in a complete medium, in serial concentrations of 2 : 1 to final concentrations of 0.002% (v/v), and 20 *μ*l of each one was added to the 96-well culture plates containing the parasite culture. After 48 h incubation, the plates were frozen (−20°C for 24 h) and thawed before starting the* p*LDH assay [40].

The hemolyzed cultures were transferred to another 96-well culture plate to which Malstat® and NBT/PES reagents were added. After 1 h of incubation at 37°C in the dark, absorbance was read at 540 nm in a spectrophotometer (Infinite®200 PRO, Tecan). The experiment was performed three times, each one in triplicate. The results were evaluated by the Microcal Origin 8.5 software to determine the dose-response curves plotted with sigmoidal fit. The IC_50_ was determined by comparison with controls for standard drug and without drugs.

#### 2.2.2. Cytotoxicity to Hep G2A16 Cells

Cytotoxicity determination was carried out in human hepatoma cell culture (Hep G2A16), which was kept at 37°C in RPMI medium supplemented with 5% fetal calf serum (complete medium) in a 5% CO_2_ environment. Cells from confluent monolayers were trypsinized, washed, counted, diluted in complete medium, distributed in 96-well microtiter plates (4 × 10^5^ cells/well), and then incubated for 18 h at 37°C. The compounds to be tested were diluted in DMSO (final concentration of 0.02%). After 24 h incubation at 37°C, 28 *µ*L of MTT solution (2 mg·mL^−1^ in PBS) was added to each well. After 1.5 h of incubation at 37°C, the supernatant was removed and 130 *µ*L of DMSO was added to each well [40, 42]. The experiment was performed three times, each one in triplicate. The culture plates were read by a spectrophotometer with a 510 nm [42]. The selective indexes (SI = CC_50_/IC_50_) of compounds** 6a–h**,** 7a–h**, and** 8a–h** were calculated.

### 2.3. Computational Methods: Molecular Docking

Initially, the molecular targets were obtained from a Protein Data Bank (PDB) [43] under codes 4PD4 (*P. falciparum *cytochrome* bc1* complex) [8] and 5FI8 (*P. falciparum *dihydroorotate dehydrogenase)*⁠* [44]. Next, the molecular target structures were represented according to the Swiss Model software [45] for building the missing loops, and the resulting structures were submitted to H++3.0 [46], a program that automates the key steps in the preparation of biomolecular structures for molecular modeling and simulations, as well as by the MGLTools *⁠*software [47] to assign the protonate state of histidines. In addition, a grid box was generated with dimensions of 20, 20, and 20 Å for both molecular targets. The coordinates of the grid box were centered on crystallographic ligands, 198.619, −25.603, and 81.313 Å for *x*, *y*, and *z*, respectively, for 4PD4, and −38.861, 102.656, and −15.221, for 5FI8, using the MGLTools software [47]. Subsequently, the HNQs and the FNQs structures were generated by the MarvinSketch*⁠* program [48]. The protonation states and the tautomers, when possible, were adjusted according to pH 7.4 [49]. Next, all compound structures were refined by the* Octopus* software*⁠* [50].* Octopus* is an automated workflow management tool that integrates MOPAC2016*⁠*, MGLTools*⁠*, PyMOL*⁠*, and AutoDock Vina to perform molecular docking through a user-friendly interface [45, 51–55]. HNQs and FNQs were refined by the parametric method 7 (PM7) [56] and the Eigenvector Following (EF) [56] routine implemented in MOPAC2016 [52] by the* Octopus *Run_mopac routine, which automatically assigns the total molecular charge for each ligand and checks for errors in the structures. The AutoDock Vina methodology was evaluated by redocking, and its exhaustiveness parameter was set to 24 to achieve more accurate results [57]. The figures were generated by academic versions of the Discovery Studio 4.5 program [58].

## 3. Results and Discussion

### 3.1. Chemistry

#### 3.1.1. Synthesis

Three series of naphthoquinones—HNQs,* ortho- *and* para-*FNQs—were synthesized, affording 24 compounds, 20 of which had been previously described [20, 29, 34], while the other 4 compounds (**6h**,** 7g**,** 7h,** and** 8h**) represent new chemical entities (Scheme 1).

The HNQs (**6a–h**) were synthesized by aldol condensation between commercial lawsone (**5**) (Sigma-Aldrich) and appropriate aldehydes, according to a methodology adapted from Hooker [59] with yields ranging from 32% to 91% (Scheme 1). The FNQs** 7a–h **and** 8a–h** were prepared by oxidative cyclization of the corresponding HNQs with Hg(OAc)_2_ [20, 34, 60], as outlined in Scheme 1. When the reaction mixtures were diluted with HCl 2N for 15 min,* ortho-*FNQs (kinetic products) were the major products, while when concentrated HCl (12N) was used with 3 h of reaction,* para-*FNQs (thermodynamic products) were obtained [60]. The* ortho-* and* para-*FNQs were obtained with yields ranging from 20% to 92% (Scheme 1). In presence of concentrated sulfuric acid,* ortho-*FNQs can be converted into* para-*FNQs, a process called isomerization, previously described by Hooker in 1986 [59]; however, this method was not used in the present work.

Some of the HNQs,* ortho-* and* para-*FNQs described in this work, had also been synthesized by our research group in prior studies, but these were only evaluated against* Toxoplasma gondii *[30–32].

A fourth series of naphthoquinones, the ANQs (**10a–c**), was prepared from lawsone (**5**) by etherification with epichlorohydrin to produce compound** 9** (58% yield), followed by a nucleophilic opening of the epoxide ring with appropriated amines affording** 10a–c** with low yields (20% to 30%), Scheme 2. All compounds of this series represent new chemical entities.

#### 3.1.2. Spectroscopic Characterization of Compounds

All the synthesized compounds were chemically characterized by their spectra in the IR (infrared), ^1^H NMR, and ^13^C NMR and some of them by HRMS and two dimensional NMR spectra. Spectra for the new chemical entities are shown in the Supplementary Data in Supplementary Material available online at https://doi.org/10.1155/2017/7496934.

### 3.2. Biological Evaluation

Initially, the percentage of parasite growth inhibition was determined for each compound in two different concentrations (50 and 25 *μ*g·mL^−1^), in triplicate, and the results are shown in Table 1. Compounds showing a parasitemia reduction greater than 50% at 25 *μ*g·mL^−1^ were classified as active.

All of the HNQs (**6a–h**),* ortho-*FNQs (**7a–h**), and* para*-FNQs (**8a–h**) were classified as* in vitro* antiplasmodial active compounds (parasitemia reduction > 50% at 25 *μ*g·mL^−1^), and their IC_50_ values were experimentally determined by the* p*LDH methodology (Table 2) [40, 42]. Lawsone glycidyl ether (**9**) and ANQs (**10a–c**) were inactive, with parasitemia reduction ranging from 9% to 34% at 25 *μ*g·mL^−1^ (Table 1); therefore, their IC_50_ and CC_50_ were not determined. It is important to highlight that the* in vitro *assays were performed in 48 h, because that is the duration of the asexual erythrocytic stage of* P. falciparum*, and it is within the time range recommended by Makler and collaborators (1998) [39] whose methodology was followed in the present* in vitro* antiplasmodial evaluations.

All of the naphthoquinones of the first three series, namely, HNQs (**6a–h**),* ortho*-FNQs (**7a–h**), and* para-*FNQs (**8a–h**), were more active than lapachol (IC_50_ 206.38 *μ*mol·L^−1^) (Table 2). The HNQs series (**6a–h**) was the least effective, with only** 6f** and** 6h** disclosing moderate activity (IC_50_ < 50 *μ*mol·L^−1^) (Table 2). In general,* ortho*-FNQs (**7a–h**) and* para*-FNQs (**8a–h**) showed higher activity (lower IC_50_) than HNQs. In total, twelve FNQs showed IC_50_ < 30 *μ*mol·L^−1^: six* ortho*-FNQs (**7a**,** 7b**,** 7c**,** 7e**,** 7f**, and** 7h**) and six* para*-FNQs (**8a**,** 8b**,** 8c**,** 8d**,** 8f**, and** 8g**), highlighting** 8c (**IC_50_ 11.65 *μ*mol·L^−1^),** 8f (**IC_50_ 18.78 *μ*mol·L^−1^), and** 7h (**IC_50_ 19.91 *μ*mol·L^−1^), which were the most active. Another interesting aspect to be noted is related to compound pairs with a phenyl and a chlorophenyl substituent in each of these three structural series. The presence of a chlorine atom was planned by analogy with atovaquone, and a surprising result was observed. For the HNQs** 6g** and** 6h** and the* ortho*-FNQs** 7g** and** 7h**, the chlorinated derivatives were about three times more active in HNQs and two times more active in* ortho*-FNQs, while for the* para*-FNQs series,** 8g** and** 8h**, an inverse effect was observed, which could, possibly, be related to the low solubility of these compounds in the conditions of the biological assays.

To investigate the selectivity of the active compounds, CC_50_ against Hep G2A16 cells was determined, and the SI values (SI = CC_50_ / IC_50_) were calculated (Table 2).

Six of the 13 less cytotoxic compounds (CC_50_ > 90 *μ*mol·L^−1^) are HNQs (**6a**,** 6b**,** 6c**,** 6e**,** 6g**, and** 6h**), while seven are* para*-FNQs (**8a**,** 8c**,** 8d**,** 8e**,** 8f**,** 8g**, and** 8h**). As expected,* ortho*-FNQs were more cytotoxic because they can be easier reduced and, therefore, more reactive oxygen species (ROS) are generated, increasing thus cell damage [61].

Nine compounds showed SI > 5: four HNQs (**6a**,** 6e**,** 6g**, and** 6h**) and five* para*-FNQs (**8a**,** 8c**,** 8d**,** 8f**, and** 8g**). Compounds** 8c **and** 8f **are highlighted here because they exhibit the best IC_50_ values (**8c** 11.65 *μ*mol·L^−1^;** 8f** 18.78 *μ*mol·L^−1^), presented low cytotoxicity (**8c **115.90 *μ*mol·L^−1^;** 8f** 97.89 *μ*mol·L^−1^), and showed SI > 5 (**8c** 9.94 and** 8f** 5.21). Despite disclosing a moderate activity, compound** 8a** (IC_50_ 26.57 *μ*mol·L^−1^) showed low cytotoxicity (CC_50_ 869.10 *μ*mol·L^−1^), resulting in the highest SI value (32.71), and might be highlighted as a lead compound, when its SI is considered; that is,** 8a** can be selected for* in vivo* antimalarial efficacy evaluation and early safety and toxicity studies. Finally, compounds** 6h**,** 8a**,** 8c**,** 8d**,** 8f,** and** 8g** showing moderate antiplasmodial activity (IC_50_ < 40 *μ*mol·L^−1^) against the chloroquine-resistant* P. falciparum *W2 strain, low cytotoxicity (CC_50_ > 90 *μ*mol·L^−1^), and SI > 5 are thus selective to resistant parasite and deserve also further investigations.

The* in vitro* antiplasmodial activity of some naphthoquinones described in this work was previously described [27, 28] as causing completely growth inhibition of an unidentified strain of* P. falciparum*. Recently, Duran-Lengua et al. (2015) [34] reported enhanced effects of** 6**,** 7**,** 8-a**,** 6**,** 7**,** 8-b**, and** 6**,** 7**,** 8-d** against sensitive and moderately resistant strains of* P. falciparum*, 3D7, and Dd2, respectively. With respect to the IC_50_ values, it should be taken into account that, in the present case, the higher IC_50_ values might be explained by the fact that the* in vitro* assays were performed upon* P. falciparum* W2, a chloroquine-resistant strain, which represents a challenge to the discovery of new antiplasmodial drugs.

### 3.3. Computational Methods: Molecular Docking

**Figure 3:**
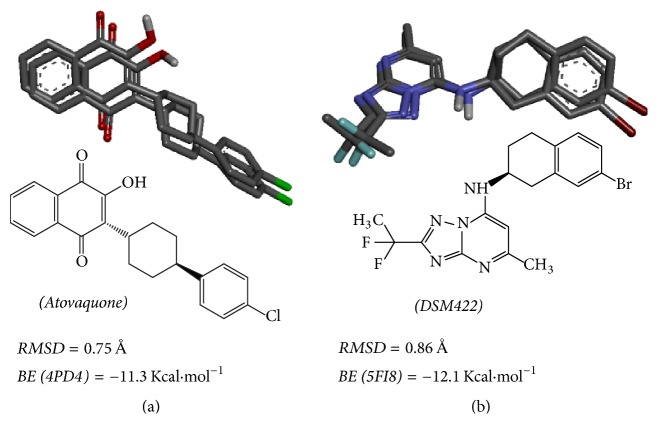
Values of the root mean square deviation (RMSD) of redocking and binding energy (BE) for the interaction of 4PD4 with atovaquone (a) and of 5FI8 with DSM422 (b).

Intermolecular interactions between HNQs and FNQs against the molecular targets 4PD4 (*Pf*cyt* bc1*) and 5FI8 (*Pf*DHOD) were simulated by the AutoDock Vina software [8, 37]. The methodology was evaluated by redocking, which consists of the docking of crystallographic structures onto the targets binding sites. This simulation can show if the docking parameters and the methodology are adequately adjusted to the system. As a result, the root mean square deviation (RMSD) of atovaquone (**2**) and DSM422 (Figure 3) redocking were 0.75 Å and 0.86 Å, respectively. These results were below the threshold value of 2.0 Å [52] and binding energies of −11.3 Kcal·mol^−1^ to atovaquone with 4PD4 and −12.1 Kcal·mol^−1^ to DSM422 with 5FI8 (Figure 3). These data suggest that the AutoDock Vina methodology is appropriate for simulating the intermolecular interaction and predicting the binding energy of HNQs and FNQs to targets (4PD4 and 5FI8).

Binding energies of docking simulations ranged from −8.2 Kcal·mol^−1^ to −10.6 Kcal·mol^−1^ for 4PD4 and from −8.2 Kcal·mol^−1^ to −11.5 Kcal·mol^−1^ for 5FI8 (Table 2). These values are relevant in the interpretation of docking results for the HNQs and FNQs, as active compounds are expected to disclose binding energies close to the models (atovaquone and DMS422) and should promote good activities.

Molecular docking simulations disclosed favorable binding energies (BE ≤ −10 Kcal·mol^−1^) for interactions of twelve compounds with the targets 4PD4 (**6g**,** 6h**,** 7g**,** 7h**,** 8g**,** 8h**) and 5FI8 (**6f**,** 6g**,** 7f**,** 8f**,** 8g**,** 8h**) which are coherent with SI > 5 for** 6g, 6h, 8f**, and** 8g**, highlighting FNQs.

**Figure 4:**
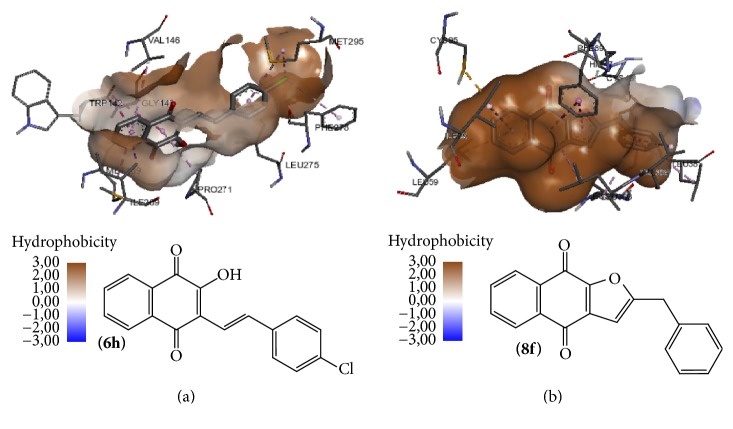
(a) Compound** 6h** complexed into the hydrophobic surface of 4PD4. (b) Compound** 8f** complexed into the hydrophobic surface of 5FI8.

For HNQ** 6d,** one of the less active compounds (SI 0.24), a weak correlation with the binding energies (4PD4 −8.5 and 5FI8 −9.0 Kcal·mol^−1^) for both of the targets (Table 2), was observed. On the other hand, for the HNQ** 6h** (SI = 8.49), the binding energy is −10.6 Kcal·mol^−1^ for 4PD4, while the value of BE fo*r para-*FNQ** 8f** (SI = 5.21) is −11.5 Kcal·mol^−1^ for 5FI8 (Table 2); these are the most active compounds of these series and their complexed structures into the hydrophobic cavities of the targets, as shown in Figure 4.

**Figure 5:**
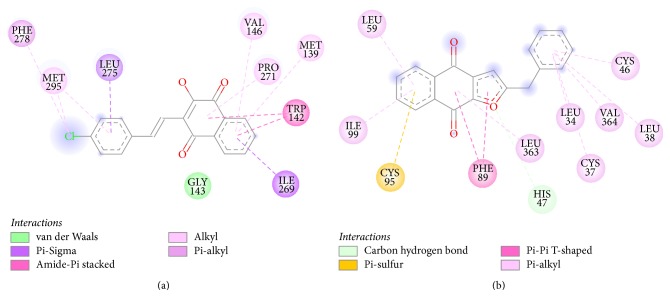
2D diagram of intermolecular interaction between** 6h** and 4PD4 (a) and between** 8f** and 5FI8 (b).

Figure 5(a) shows that HNQ** 6h** represents van der Waals intermolecular interactions with 4PD4 through amino acids Met139, Gly143, Val146, Trp142, Ile269, Pro271, Leu275, and Phe278. Similarly, for compound** 8f,** van der Waals interactions are depicted with Leu34, Cys37, Leu38, Leu59, Cys46, Phe89, Ile99, Leu363, and Leu364 amino acids of 5FI8 (Figure 5(b)). In addition, the molecular recognition for compound** 8f** is improved by two weak noncovalent intermolecular interactions: His47 and the furan oxygen with a distance of 2.69 Å and a *π*-sulfur interaction between Cys95 and the benzenoid ring in the naphthoquinone moiety with a distance of 2.47 Å. These intermolecular interactions might certainly contribute for the biological activity of compounds** 6h** and** 8f**.

Previous studies have shown the inhibition of* Pf*cyt* bc1* and* Pf*DHOD by naphthoquinones [8, 10, 11], and the molecular docking results described here are in accordance with this mode of action, although further enzymatic assays are necessary for confirmation.

## 4. Conclusions

The present study reported on the synthesis of 28 compounds belonging to four structural series—HNQs,* ortho-*FNQs,* para-*FNQs, and ANQs—eight of which represented new chemical entities. All of the HNQs (**6a–h**) and FNQs (**7a–h** and** 8a–h**) were classified as active compounds with* in vitro* parasitemia reduction ranging from 45% to 90% at 25 *μ*g·mL^−1^ and proved to be more potent than lapachol (IC_50_ 206.38 *μ*mol·L^−1^) against the chloroquine-resistant* P. falciparum* W2 strain. The insertion of a furan ring in the naphthoquinone moiety increased the biological activity, and the FNQs were, in general, more active than the HNQs. As expected,* ortho*-FNQs (**7a–h**) were more cytotoxic to Hep G2A16 cells and less selective to the parasite (SI < 5). The* ortho*-FNQs might be highlighted for their potential antitumoral rather than antiplasmodial effect. On the other hand, ANQs, although containing an amino group in their structures, disclosed low biological activity and were classified as inactive compounds (parasitemia reduction < 50% at 25 *μ*g·mL^−1^). Considering the experimental values of IC50 < 40 *μ*g·mL^−1^, CC50 > 90 *μ*g·mL^−1^, and SI > 5, the* para*-FNQ** 8a** is highlighted for its high value of SI (32.71). However, the following naphthoquinones** 6h**,** 8c**,** 8d**,** 8f,** and** 8g** deserve also further investigations. Molecular docking simulations disclosed most favorable binding energies (<−10 Kcal·mol^−1^) for** 6h** and** 8f**, disclosing good correlations with the present biological data for naphthoquinones that support aryl moieties. This study points out to arylnaphthoquinones as new potential inhibitors of the* Pf*cyt* bc1* and* Pf*DHOD enzymes since, for both of these targets, favorable binding energies were calculated. However enzymatic assays are necessary for confirming this hypothesis.

## Supplementary Material

Copies of ^1^H and ^13^C NMR and HRMS spectra can be viewed in the Supplementary Information.

## Figures and Tables

**Table 1:** Percentages of *Plasmodium falciparum* (W2) parasitemia reduction at 50 *μ*g·mL^−1^ and 25 *μ*g·mL^−1^ for naphthoquinones **6a–h**, **7a–h**, **8a–h**, **9**, and **10a–c**.

Compound	% reduction at 50 *μ*g·mL^−1^	% reduction at 25 *μ*g·mL^−1^
**6a**	75	53
**6b**	73	80
**6c**	64	50
**6d**	91	81
**6e**	71	53
**6f**	64	54
**6g**	78	73
**6h**	83	58
**7a**	98	98
**7b**	90	81
**7c**	94	91
**7d**	81	81
**7e**	94	88
**7f**	81	75
**7g**	79	18
**7h**	95	89
**8a**	83	75
**8b**	85	78
**8c**	92	86
**8d**	71	70
**8e**	83	76
**8f**	87	85
**8g**	80	78
**8h**	62	24
**9**	61	34
**10a**	21	9
**10b**	22	16
**10c**	15	12

**Table 2:** * In vitro* antiplasmodial activity (IC_50_) against *Plasmodium falciparum* (W2), cytotoxicity (CC_50_, Hep G2A16 cells), selectivity index (SI), and binding energy values of docking simulations for 4PD4 and 5FI8 with naphthoquinones **6a–h**, **7a–h**, and **8a–h**.

Compound	IC_50_ (*μ*mol·L^−1^)^b^	CC_50_ (*μ*mol·L^−1^)^c^	SI^d^	Binding energy 4PD4^e^ (Kcal·mol^−1^)	Binding energy 5FI8^f^ (Kcal·mol^−1^)
**6a**	99.85 ± 8.46	970.00 ± 42.60	9.72	**−**8.2	−8.2
**6b**	59.24 ± 10.27	138.97 ± 15.14	2.34	−8.5	−8.5
**6c**	52.65 ± 3.71	146.80 ± 29.10	2.79	−8.5	−8.7
**6d**	90.17 ± 10.43	21.55 ± 9.44	0.24	−8.5	−9.0
**6e**	72.21 ± 2.98	1,127.00 ± 39.67	15.61	−8.9	−9.0
**6f**	45.76 ± 2.55	19.51 ± 4.03	0.43	−9.4	−10.2
**6g**	110.34 ± 6.64	940.30 ± 40.10	8.52	−10.3	−10.7
**6h**	39.55 ± 8.98	335.90 ± 26.70	8.49	−10.6	−9.1
**7a**	23.22 ± 5.09	4.68 ± 0.70	0.20	−8.6	−9.1
**7b**	25.10 ± 2.64	11.91 ± 4.65	0.47	−8.6	−9.1
**7c**	24.70 ± 2.25	11.53 ± 2.97	0.47	−8.5	−9.2
**7d**	50.22 ± 2.90	12.99 ± 2.01	0.26	−8.6	−9.3
**7e**	26.41 ± 3.12	21.33 ± 3.15	0.80	−9.2	−9.3
**7f**	23.44 ± 6.39	13.68 ± 4.46	0.58	−9.9	−10.2
**7g**	40.72 ± 7.82	6.10 ± 0.84	0.15	−10.2	−9.9
**7h**	19.91 ± 4.35	68.68 ± 14.79	3.45	−10.0	−9.8
**8a**	26.57 ± 3.25	869.10 ± 32.71	32.71	−8.2	−8.9
**8b**	28.86 ± 3.66	12.51 ± 2.18	0.43	−8.4	−9.0
**8c**	11.65 ± 2.50	115.90 ± 21.90	9.94	−8.3	−9.1
**8d**	23.77 ± 2.41	119.20 ± 23.70	5.01	−8.4	−9.5
**8e**	30.55 ± 3.81	143.27 ± 12.24	4.69	−8.9	−9.4
**8f**	18.78 ± 0.83	97.89 ± 13.22	5.21	−9.90	−11.5
**8g**	21.80 ± 2.64	120.84 ± 6.05	5.54	−10.0	−11.3
**8h**	140.70 ± 7.80	1,048.90 ± 32.90	2.40	−10.3	−10.5
**2**	206.38 ± 3.64	1567.96 ± 7.44	7.6	ND	ND
^a^CQ	0.280 ± 0.01	361.84 ± 10.64	1,292.30	ND	ND

^a^CQ: chloroquine; ^b^IC_50_: concentration (*μ*mol·L^−1^) that kills 50% of *P. falciparum*, determined by the *p*LDH method; ^c^CC_50_: concentration that kills 50% of Hep G2A16 cells 24 h after incubation with the compounds, determined by the MTT method; ^d^SI (selectivity index): CC_50_/IC_50_; ^e^4PD4: cytochrome bc1 complex subunit 1; ^f^5FI8: dihydroorotate dehydrogenase; ND: not determined.
