# Supplementary material for: Synthesis, SAR, and Docking Studies Disclose 2-Arylfuran-1,4-naphthoquinones as In Vitro Antiplasmodial Hits
Source: J Trop Med. 2017 Oct 31;2017:7496934. doi: 10.1155/2017/7496934 (PMC5684547; doi:10.1155/2017/7496934)
Supplement: Supplementary file 1 — Copies of 1H and 13C NMR and HRMS spectra can be viewed in the Supplementary Information. [file 7496934.f1.pdf]

Supplementary Information

($E$)-2-(4-chlorostyryl)-3-hydroxynaphthalene-1,4-dione (**6h**)

Yield: 91%; red solid; MP: 195.5-195.9 $^0$C; IR cm$^{-1}$: 3254 (O-H), 2956 (C-H$_{aromatic}$), 1664 and 1645 (C=O), 1274 (C-O), 723 (C-Cl); $^1$H- NMR (400 MHz, $CDCl_3$) $\delta$: 8.16 (dd, 1H, $J_{5,6}$= 7.66 Hz, $J_{5,7}$= 0.70 Hz, H-5), 8.11 (dd, 1H, $J_{8,7}$= 8.11 Hz, $J_{8,6}$= 0.84 Hz, H-8), 7.78 (t, 1H, $J_{6,7 \text{ and } 6,5}$= 7.58 Hz, H-6), 7.71 (t, 1H, $J_{7,6 \text{ and } 7,8}$= 7.58 Hz, H-7), 7.99 (s, 1H, OH), 7.38-7.34 (m, 3H, H-1' and H-5'), 7.94 (d, 1H, $J_{2',1'}$= 16.68 Hz, H-2'), 7.53 (d, 2H, $J_{5',4'}$= 8.44 Hz, H-4'); $^{13}$C- NMR (100 MHz, $CDCl_3$) $\delta$: 181.36 (C-1), 152.26 (C-2), 118.65 (C-3), 184.31 (C-4), 127.45 (C-5), 135.34 (C-6), 133.50 (C-7), 126.39 (C-8), 129.81 (C-9), 132.99 (C-10), 118.24 (C-1'), 138.04 (C-2'), 136.59 (C-3'), 128.40 (C-4'), 129.15 (C-5'), 134.59 (C-6'); HRMS-ESI (m/z): [M-H$^+$] calcd for $C_{18}H_{12}O_3$: 275.0708, found: 275.0677, error 11 ppm.

2-Phenylnaphtho[1,2-$b$]furan-4,5-dione (**7g**)

Yield: 30 %; red solid; MP: 214.5-217.0 $^0$C; IR cm$^{-1}$: 3069 (C-H$_{aromatic}$), 2921 (C-H), 1668 (C=O); $^1$H- NMR (400 MHz, $CDCl_3$) $\delta$: 7.73-7.41 (m, 2H, H-5 and H-6), 7.67 (t, 1H, $J_{7,8 \text{ and } 7,6}$=7.50 Hz, H-7), 8.06 (d, 1H, $J_{8,7}$=7.68 Hz, H-8), 7.01 (s, 1H, H-1'), 7.48-7.36 (m, 4H, H-4' and H-5'), 7.78-7.77 (m, 1H, H-6'); $^{13}$C- NMR (100 MHz, $CDCl_3$) $\delta$: 180.55 (C-1), 174.62 (C-2), 123.51 (C-3), 156.94 (C-4), 122.41 (C-5), 135.63 (C-6), 129.42 (C-7), 130.39 (C-8), 129.08 (C-9), 128.84 (C-10), 103.02 (C-1'), 159.91 (C-2'), 128.63 (C-3'), 129.21 (C-4'), 124.61 (C-5'), 130.80 (C-6').

2-(4-Chlorophenyl)naphtho[1,2-$b$]furan-4,5-dione (**7h**)

Yield: 20%; red solid; MP: 284.1-284.8.0 $^0$C; IR cm$^{-1}$: 3245 (C-H$_{aromatic}$), 2921 (C-H), 1704 and1668 (C=O); $^1$H- NMR (400 MHz, $CDCl_3$) $\delta$: 8.11 (d, 1H, $J_{8,7}$=7.68 Hz, H-8), 7.81 (d, 1H, $J_{5,6}$=7.60 Hz, H-5), 7.71 (s, 1H, H-7), 7.49 (t, 1H, $J_{6,7 \text{ and } 6,5}$=7.72 Hz, H-6), 7.05 (s, 1H, H-1'), 7.69-7.67 (m, 2H, H-4'), 7.48-7.44 (m, 2H, H-5'); $^{13}$C- NMR (100 MHz, $CDCl_3$) $\delta$: 180.57 (C-1), 174.64 (C-2), 123.56 (C-3), 155.91 (C-4), 122.51 (C-5), 135.45 (C-6), 130.61 (C-7), 130.97 (C-8), 129.57 (C-9), 128.55 (C-10), 103.66 (C-1'), 160.16 (C-2'), 125.93 (C-3'), 125.71 (C-4'), 129.35 (C-5'), 135.70 (C-6').

2-(4-Chlorophenyl)naphtho[2,3-*b*]furan-4,9-dione (**8h**)

Yield: 30% ; orange solid; MP: 255.8-258.7 $^0$C; IR cm$^{-1}$: 3113 (C-H$_{aromatic}$), 2922 (C-H), 1665 (C=O); $^1$H- NMR (400 MHz, $CDCl_3$) $\delta$: 8.12 (d, 1H, $J_{8,7}$=7.62 Hz, H-8), 7.91 (d, 1H, $J_{5,6}$=7.64 Hz, H-5), 7.72 (s, 1H, H-7), 7.60 (t, 1H, $J_{6,7 \text{ and } 6,5}$=7.72 Hz, H-6), 7.06 (s, 1H, H-1'), 7.70-7.68 (m, 2H, H-4'), 7.49-7.44 (m, 2H, H-5'); $^{13}$C- NMR (100 MHz, $CDCl_3$) $\delta$: 181.57 (C-1), 172.64 (C-2), 123.54 (C-3), 155.81 (C-4), 122.71 (C-5), 135.45 (C-6), 130.61 (C-7), 130.97 (C-8), 129.57 (C-9), 128.55 (C-10), 103.66 (C-1'), 160.17 (C-2'), 125.89 (C-3'), 125.71 (C-4'), 129.34 (C-5'), 135.69 (C-6').

2-(Oxiran-2-ylmethoxy)naphthalene-1,4-dione (**9**)

Yield: 58 %; yellow solid; MP: 97-100 $^0$C; IR cm$^{-1}$: 2973 (C-H$_{aromatic}$), 1651 (C=O), 1115 (C-O $_{alcohol}$), 1198 (C-O $_{epoxide}$), 1098 (C-O $_{ether}$); $^1$H- NMR (400 MHz, $CDCl_3$) $\delta$: 7.70 (d, 1H, $J_{8,7}$= 7.40 Hz, H-8), 7.62 (t, 1H, $J_{6,7 \text{ and } 6,8}$= 7.20 Hz, H-6), 8.08 (d, 1H, $J_{5,6}$= 7.52 Hz, H-5), 7.53 (t, 1H, $J_{7,8 \text{ and } 7,6}$= 7.20 Hz, H-7), 5.74 (s, 1H, H-3), 4.92 (bs, 1H, H-12), 4.50 (d, 1H, $J_{11a,11b}$= 11.50 Hz, H-11a), 4.24 (d, 1H, $J_{11b,11a}$= 11.50 Hz, H-11b), 4.41 (d, 1H, $J_{13a,13b}$= 6.64 Hz, H-13a), 4.30-4.27 (m, 1H, H-13b); $^{13}$C- NMR (100 MHz, $CDCl_3$) $\delta$: 184,52 (C-1), 106.32 (C-2), 96.84 (C-3), 165.78 (C-4), 130.44

(C-5), 132.80 (C-6), 126.15 (C-7), 126.29 (C-8), 135.03 (C-9), 132.86 (C-10), 74.47 (C-11), 72.47 (C-12), 68.14 (C-13); HRMS-ESI (m/z): $[M+Na]^+$ calcd for $C_{13}H_{10}O_4$: 253.0477, found: 253.0482, error 2ppm.

2-(3-(Ethylamino)-2-hydroxypropoxy)naphthalene-1,4-dione (**10a**)

Yield: 30 %; white solid; MP: 209.9-210.6 $^0$C; IR $cm^{-1}$: 3225 (OH); 3057 (NH); 2943 (C-$H_{aromatic}$), 1598 (C=O), 1117 (C-O $_{alcohol}$), 1093 (C-O $_{ether}$); $^1$H- NMR (400 MHz, $CDCl_3$) $\delta$: 7.86-7.84 (m, 2H, H-8 and H-5), 7.49-7.54 (m, 2H, H-6 and H-7), 5.16 (s, 1H, H-3), 3.98-3.94 (t, m, H-13a), 4.15 (t, 1H, $J_{11b,11a \text{ and } 11b,12}$= 8.12 Hz, H-11b), 4.79 (t, 1H, $J_{12,11a \text{ and } 12,11b}$ = 7.30, H-12), 3.67 (dd, 1H, $J_{13b,NH}$ = 3.04 Hz and $J_{13b,13a}$= 12.20 Hz, H-13b), 3.09 (t, 2H, $J_{14,15}$ = 6.72 Hz, H-14), 1.19 (t, 2H, $J_{15,14}$ = 7.24, Hz, H-15); $^{13}$C- NMR (100 MHz, $CDCl_3$) $\delta$: 179.96 (C-1), 101.01 (C-2), 94.29 (C-3), 161.38 (C-4), 128.95 (C-5), 131.15 (C-6), 124.76 (C-7), 125.04 (C-8), 140.00 (C-9), 131.60 (C-10), 64.88 (C-11), 79.66 (C-12), 58.5 (C-13), 37.08 (C-14), 12.98 (C-15); HRMS-ESI (m/z): $[M-CH_2CH_3]^+$ calcd for $C_{17}H_{21}NO_4$: 274.1080, found: 274.1000, error 29 ppm.

2-(2-Hydroxy-3-(isopropylamino)propoxy)naphthalene-1,4-dione (**10b**)

Yield: 20 %; white solid; MP: 242-244 $^0$C; IR $cm^{-1}$: 3224 (OH); 3062 (NH); 2980 (C-$H_{aromatic}$), 1600 (C=O), 1117 (C-O $_{alcohol}$), 1093 (C-$O_{ether}$); $^1$H- NMR (400 MHz, $CDCl_3$) $\delta$: 7.87-7.84 (m, 1H, H-8), 7.91-7,89 (m, 1H, H-5), 7.54 (d, 2H, $J_{6,7-6,5 \text{ and } 7,8-7,6}$= 7.60 Hz, H-7 and H-6), 4.02-3.98 (m, 1H, H-13a), 3.68 (dd, 1H, $J_{13b,13a}$= 12.08 Hz, $J_{13b,NH}$= 33.72 Hz, H-13b), 4.79 (t, 1H, $J_{12,11a \text{ and } 12,13b}$ = 7.26, H-12), 4.26 (t, 1H, $J_{11a,12 \text{ and } 11a,11b}$= 7.44 Hz, H-11a), 4.14 (t, 1H, $J_{11b,12 \text{ and } 11b,11a}$= 8.22 Hz, H-11b), 3.64-3.59 (m, 1H, H-14), 1.17 (d, 3H, $J_{15,14}$= 6.44 Hz, H-15), 1.15 (d, 3H, $J_{16,14}$= 6.44 Hz, H-16); $^{13}$C- NMR (100 MHz, $CDCl_3$) $\delta$: 179.92 (C-1), 101.00 (C-2), 94.21 (C-3),

160.51 (C-4), 128.92 (C-5), 131.07 (C-6), 124.70 (C-7), 124.93 (C-8), 139.95 (C-9), 131.68 (C-10), 64.88 (C-11), 79.88 (C-12), 58.96 (C-13), 43.45 (C-14), 21.40 (C-15), 21.24 (C-16); HRMS-ESI (m/z): $[M+H]^+$ calcd for $C_{16}H_{19}NO_4$: 290.1392, found: 290.1396, error 1.37 ppm.

2-(3-(Benzylamino)-2-hydroxypropoxy)naphthalene-1,4-dione (**10c**)

Yield: 25 %; white solid; MP: 180.20-181.70 $^0$C; IR cm$^{-1}$: 3225 (OH); 3057 (NH); 2943 (C-H$_{aromatic}$), 1598 (C=O), 1119 (C-O $_{alcohol}$), 1151 (C-O $_{ether}$); $^1$H- NMR (400 MHz, $CDCl_3$) $\delta$: $^1$H- NMR (400 MHz, $CDCl_3$) $\delta$: 7.83 (d, 1H, $J_{8,7}$= 8.00 Hz, H-8), 8.29 (s, 1H, H-5), 7.58-7.53 (m, 1H, H-6), 7.47 (t, 1H, $J_{7,6 \text{ and } 7,8}$= 7.00 Hz, H-7), 5.17 (s, 1H, H-3), 4.40-4.38 (m, 3H, H-11a and H-14), 3.69 (d, 1H, $J_{13b,13a}$= 12.04 Hz, H-13b), 4.77 (t, 1H, $J_{12,11a \text{ and } 12,11b}$= 7.44 Hz, H-12), 4.23 (t, 1H, $J_{11b,11a}$= 8.00, H-13b), 7.35-7.34 (m, 5H, H-16-18); $^{13}$C- NMR (100 MHz, $CDCl_3$) $\delta$: 180.13 (C-1), 101.31 (C-2), 95.50 (C-3), 161.20 (C-4), 128.91 (C-5), 131.17 (C-6), 124.80 (C-7), 124.86 (C-8), 140.06 (C-9), 131.39 (C-10), 65.10 (C-11), 79.39 (C-12), 59.71 (C-13), 45.35 (C-14), 137.44 (C-15), 126.97 (C-16), 128.48 (C-17), 127.07 (C-18); HRMS-ESI.

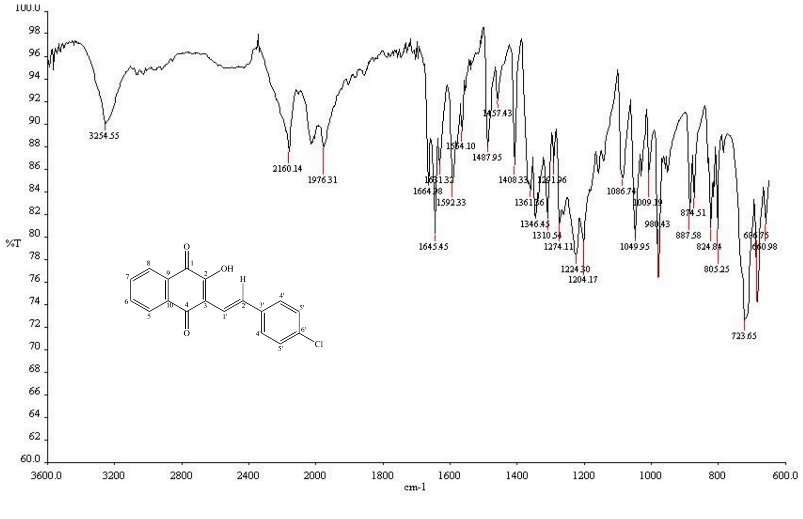


**Figure S1:** IR (ATR) spectrum of (*E*)-2-(4-chlorostyryl)-3-hydroxynaphthalene-1,4-dione (**6h**).

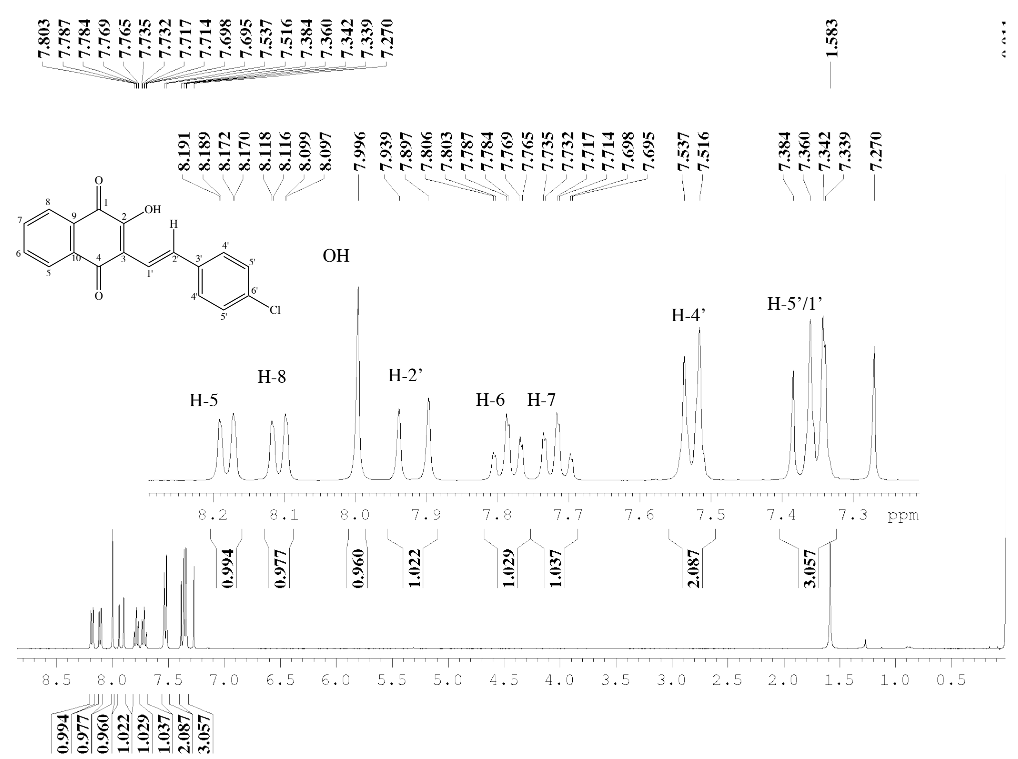

**Figure S2:** $^{1}H$ NMR spectrum (400 MHz, $CDCl_3$) of (*E*)-2-(4-chlorostyryl)-3-hydroxynaphthalene-1,4-dione (**6h**).

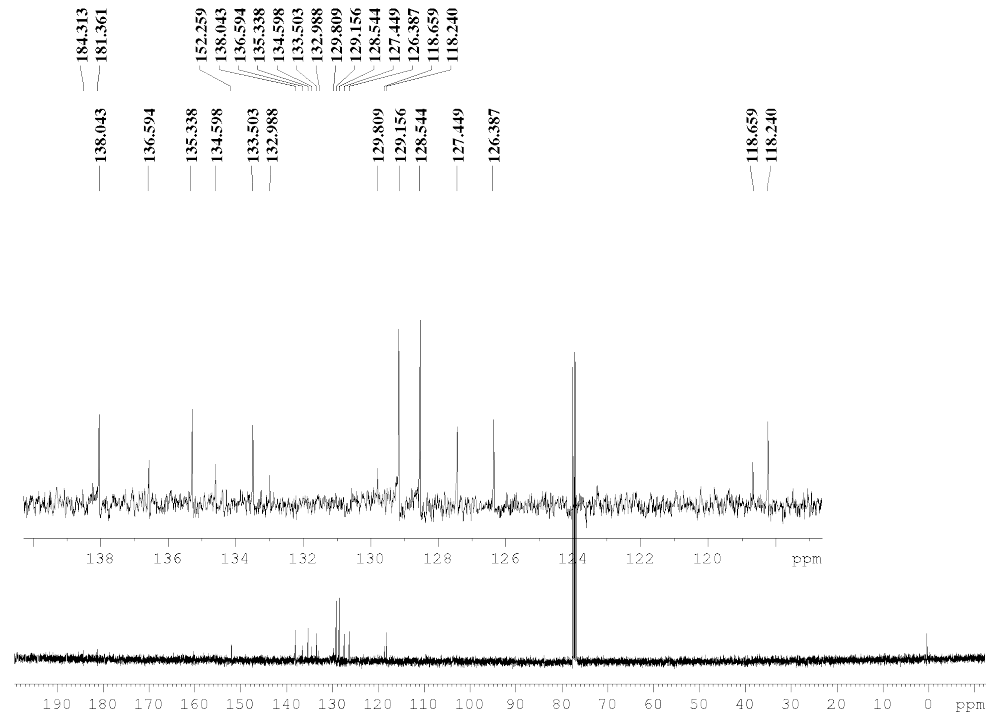

**Figure S3:** $^{13}C$ NMR spectrum (100 MHz, $CDCl_3$) and DEP 135 of (*E*)-2-(4-chlorostyryl)-3-hydroxynaphthalene-1,4-dione (**6h**).

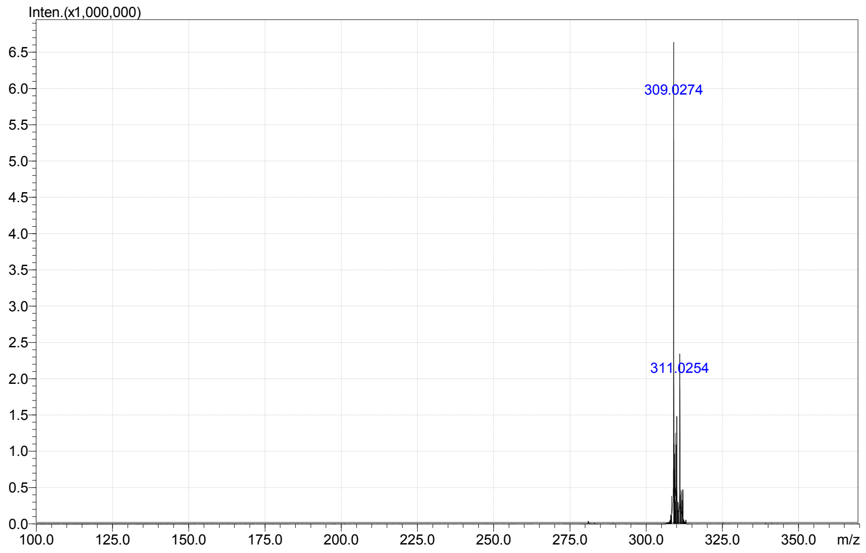


**Figure S4:** HRMS spectrum of of (*E*)-2-(4-chlorostyryl)-3-hydroxynaphthalene-1,4-dione (**6h**).

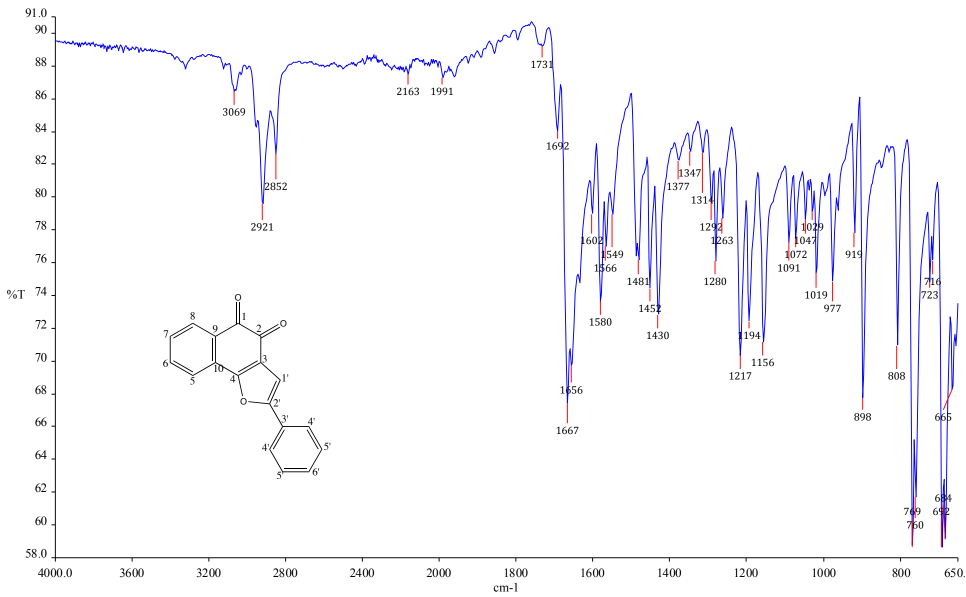


**Figure S5:** $^1$H NMR spectrum (400 MHz, $CDCl_3$) of 2-phenylnaphtho[1,2-*b*]furan-4,5-dione (**7g**).

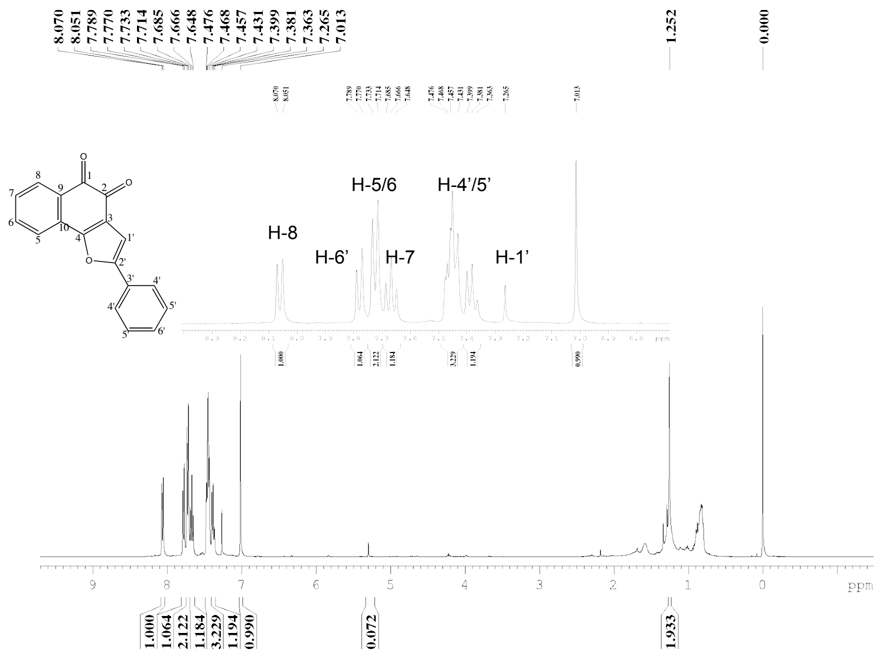


**Figure S6:** $^{1}$H NMR spectrum (400 MHz, $CDCl_3$) of 2-phenylnaphtho[1,2-*b*]furan-4,5-dione (**7g**).

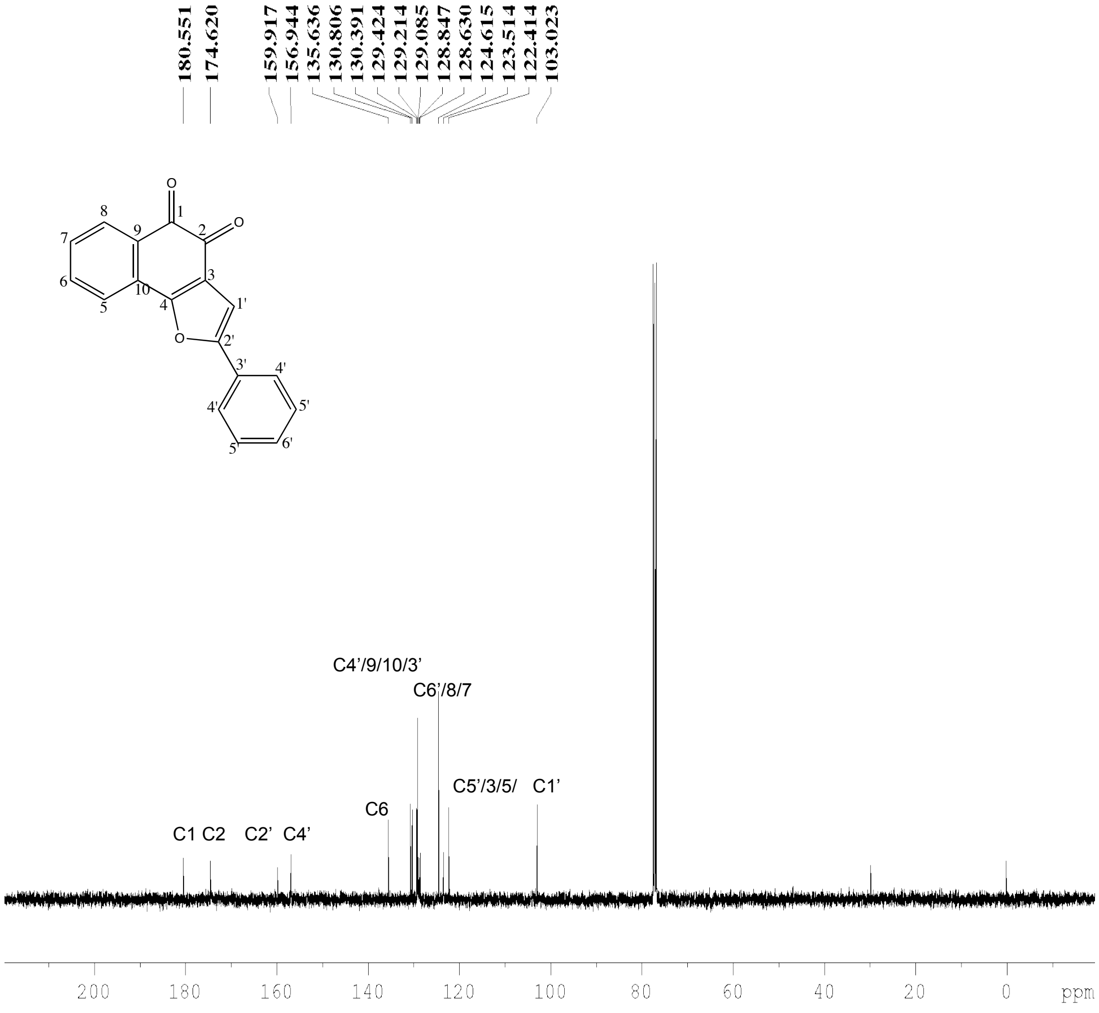


**Figure S7:** $^{13}$C NMR spectrum (100 MHz, $CDCl_3$) of 2-phenylnaphtho[1,2-*b*]furan-4,5-dione (**7g**).

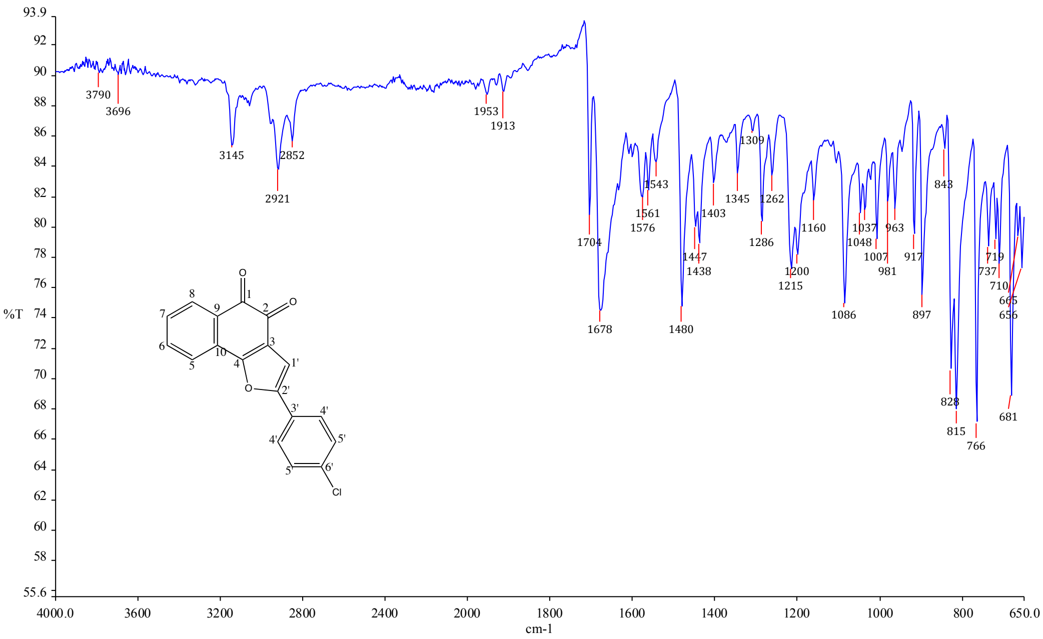

**Figure S8:** IR (ATR) spectrum of 2-(4-chlorophenyl)naphtho[1,2-*b*]furan-4,5-dione (**7h**).

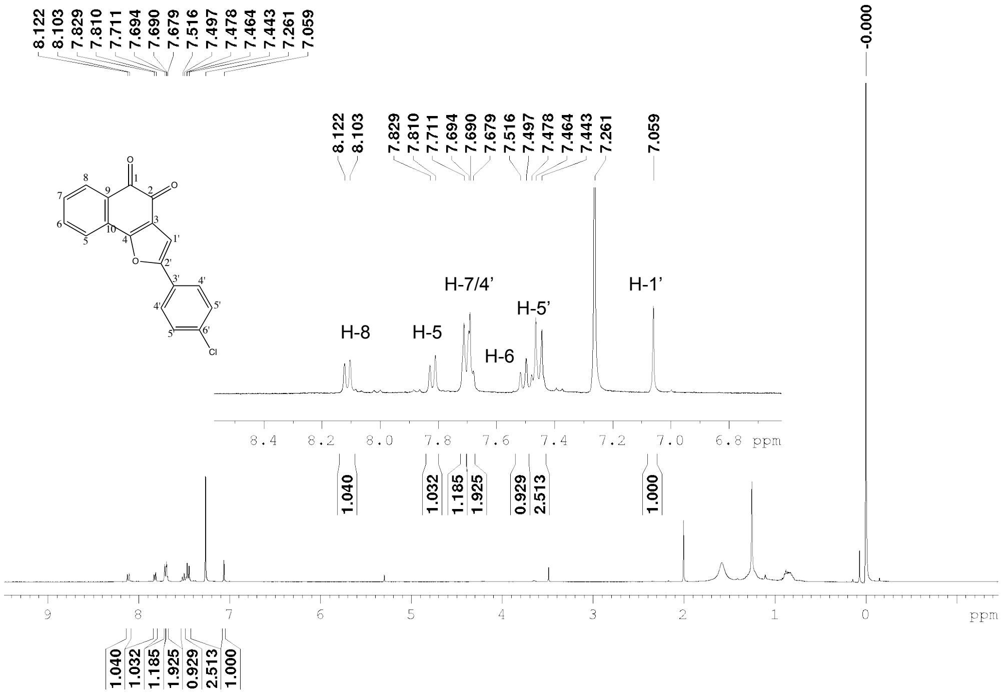

**Figure S9:** $^{1}H$ NMR spectrum (400 MHz, $CDCl_3$) of 2-(4-chlorophenyl)naphtho[1,2-*b*]furan-4,5-dione (**7h**).

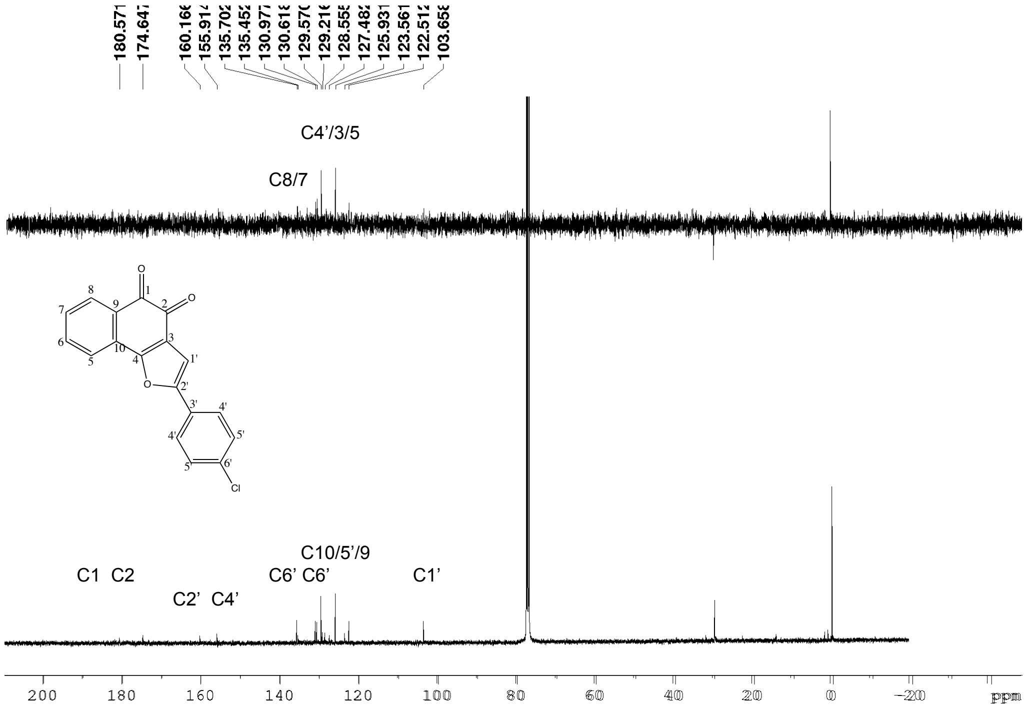


**Figure S10:** $^{13}C$ NMR spectrum (100 MHz, $CDCl_3$) and DEPT 135 of 2-(4-chlorophenyl)naphtho[1,2-*b*]furan-4,5-dione (**7h**).

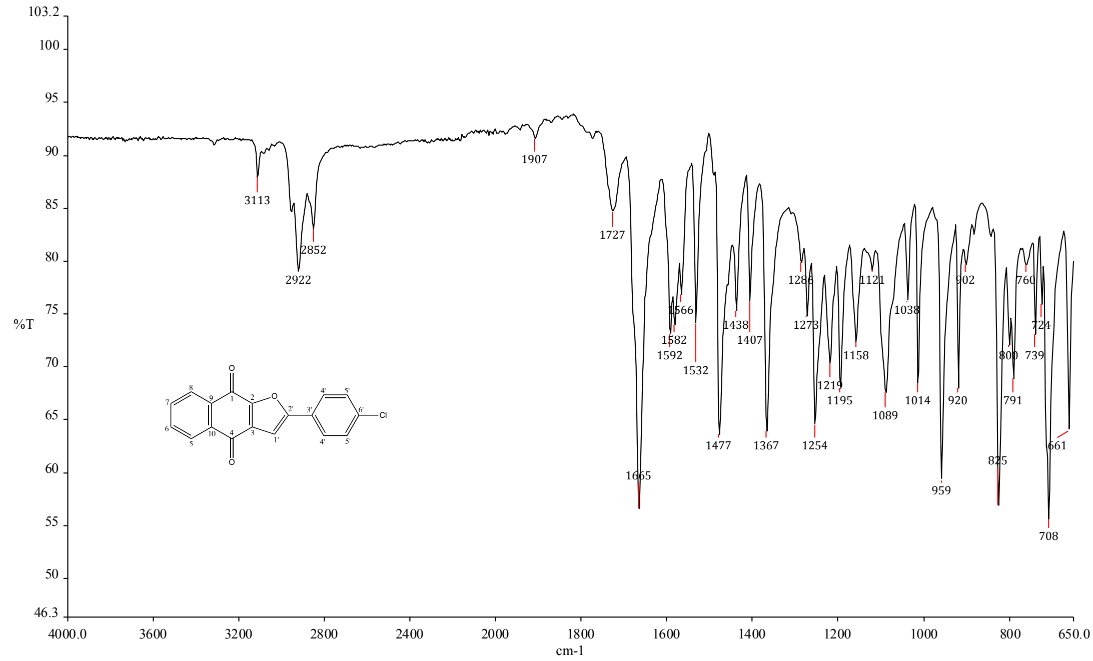


**Figure S11:** IR (ATR) spectrum of 2-(4-chlorophenyl)naphtho[2,3-*b*]furan-4,9-dione (**8h**).

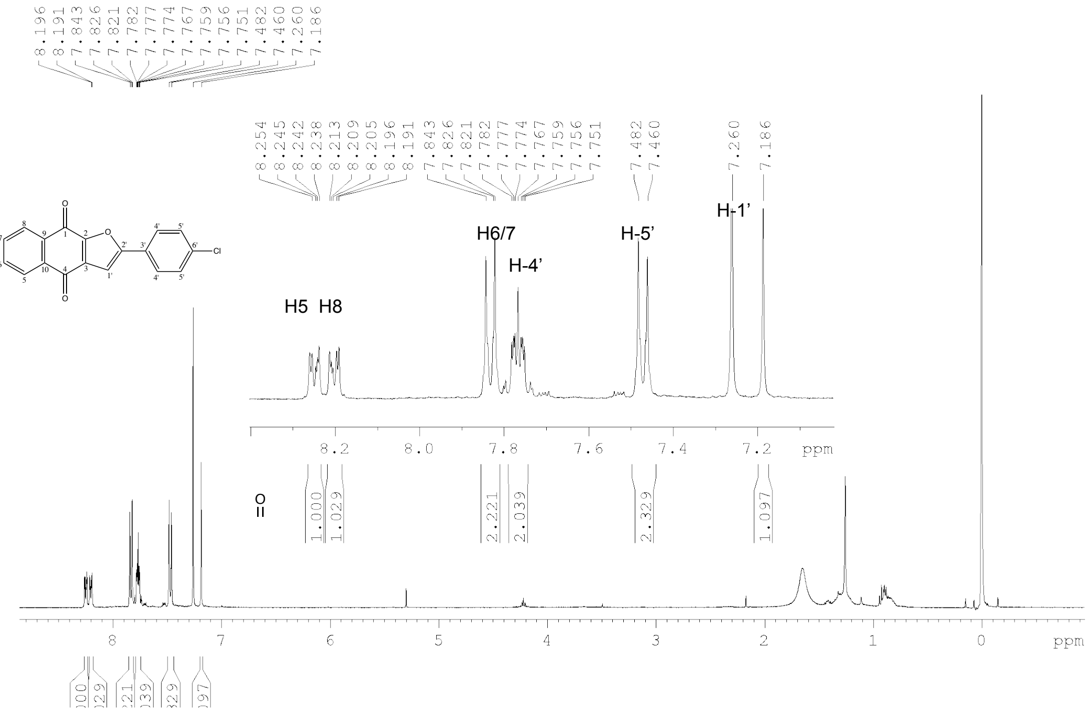

**Figure S12:** $^{1}$H NMR spectrum (400 MHz, $CDCl_3$) of 2-(4-chlorophenyl)naphtho[2,3-*b*]furan-4,9-dione (**8h**).

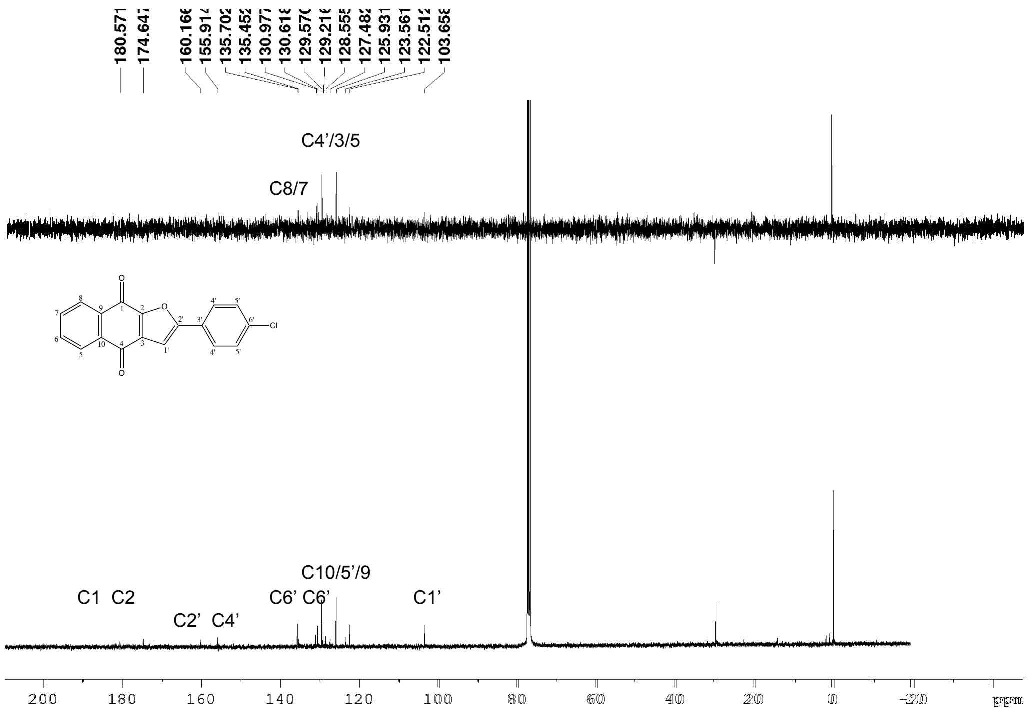

**Figure S13:** $^{13}$C NMR spectrum (100 MHz, $CDCl_3$) and DEPT 135 of 2-(4-chlorophenyl)naphtho[2,3-*b*]furan-4,9-dione (**8h**).

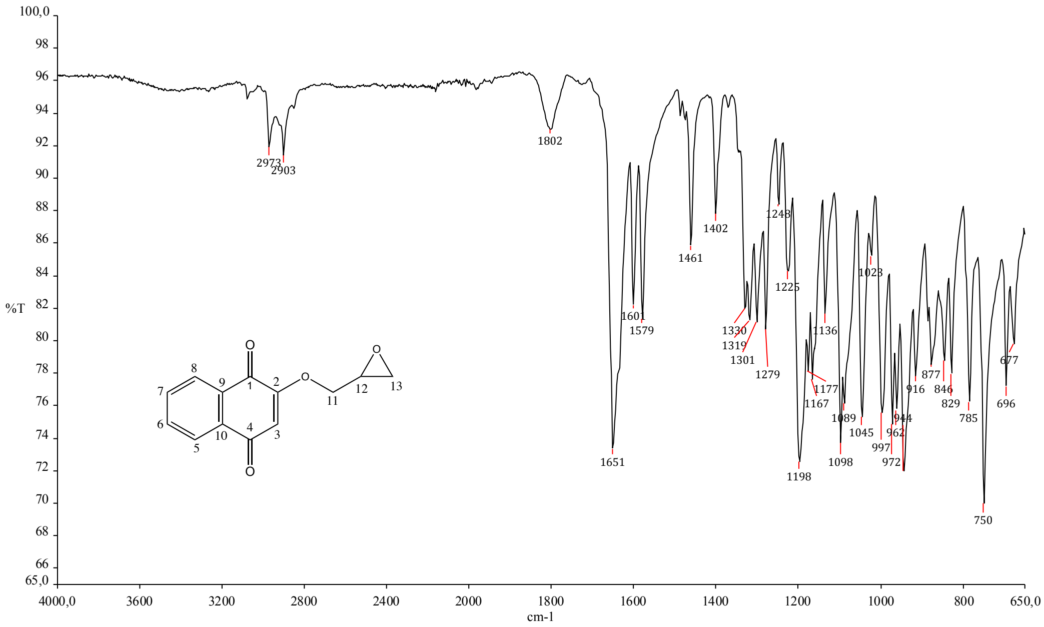


**Figure S14:** IR (ATR) spectrum of 2-(oxiran-2-ylmethoxy)naphthalene-1,4-dione (**9**).

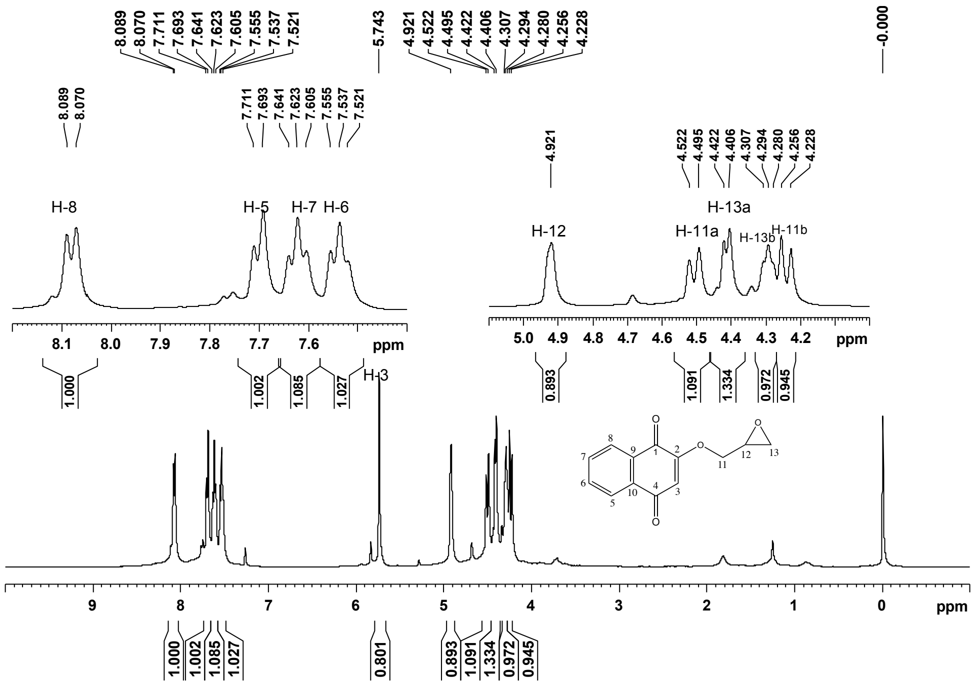


**Figure S15:** $^1$H NMR spectrum (400 MHz, DMSOd6) of 2-(oxiran-2-ylmethoxy)naphthalene-1,4-dione (**9**).

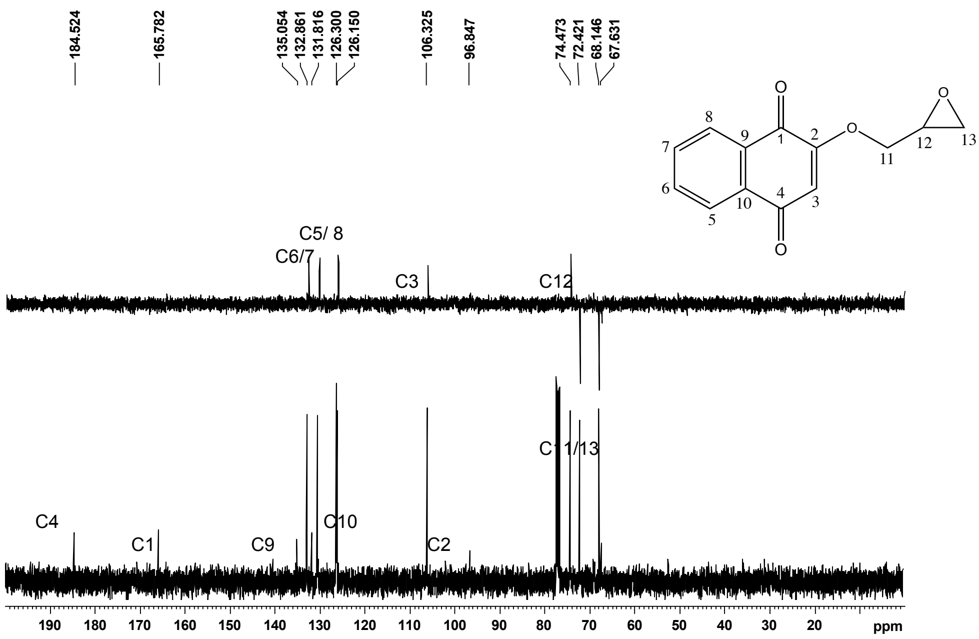


**Figure S16:** $^{13}$C NMR spectrum (100 MHz, DMSOd6) and DEPT 135 of 2-(oxiran-2-ylmethoxy)naphthalene-1,4-dione (**9**).

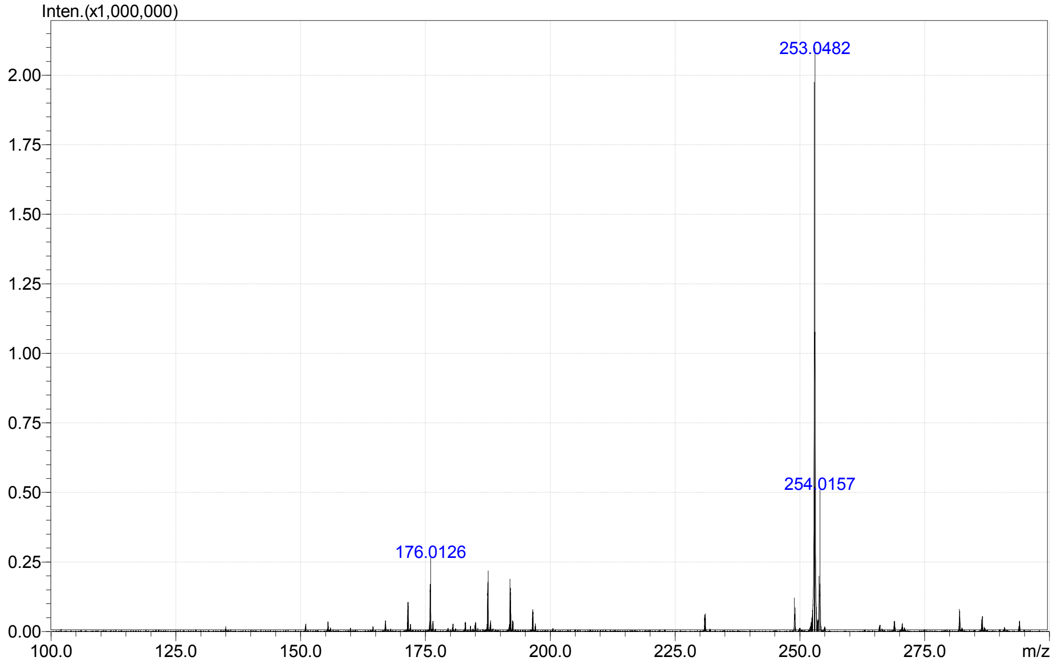


**Figure S17:** HRMS spectrum of 2-(oxiran-2-ylmethoxy)naphthalene-1,4-dione (**9**).

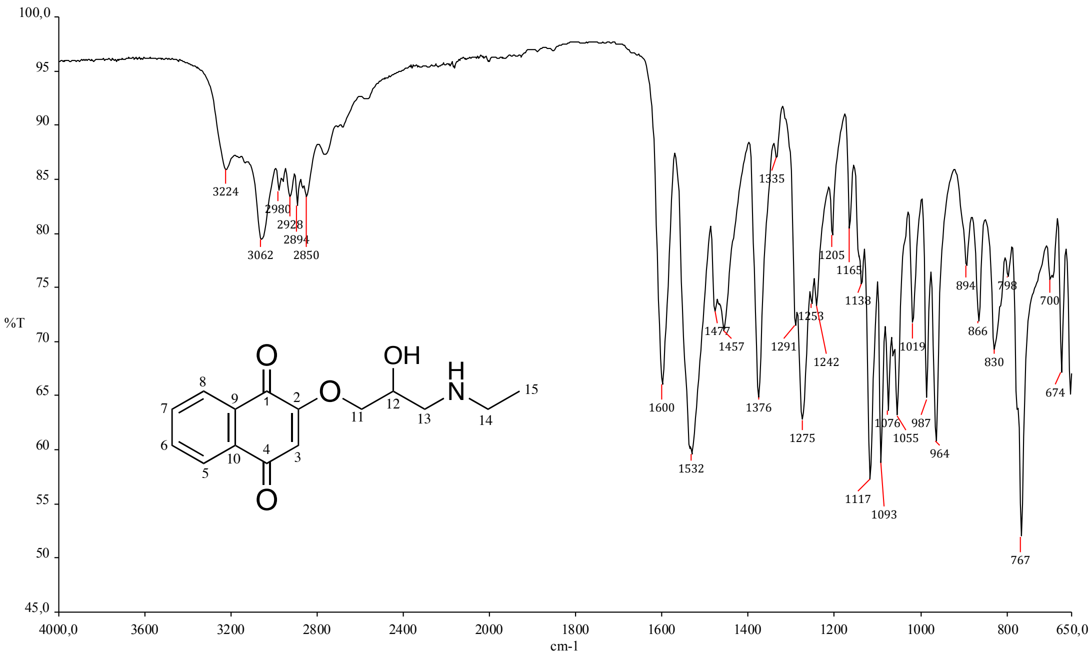


**Figure S18:** IR (ATR) spectrum of 2-(3-(ethylamino)-2-hydroxypropoxy)naphthalene-1,4-dione (**10a**).

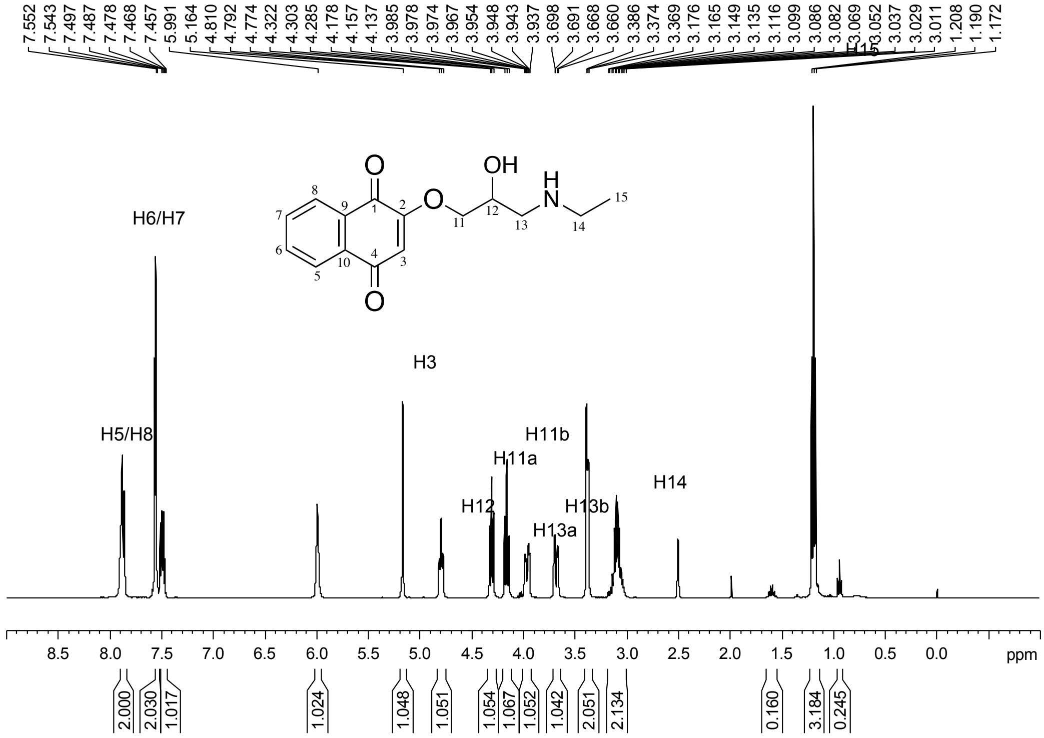


**Figure S19:** $^{1}$H NMR spectrum (400 MHz, DMSOd6) of 2-(3-(ethylamino)-2-hydroxypropoxy)naphthalene-1,4-dione (**10a**).

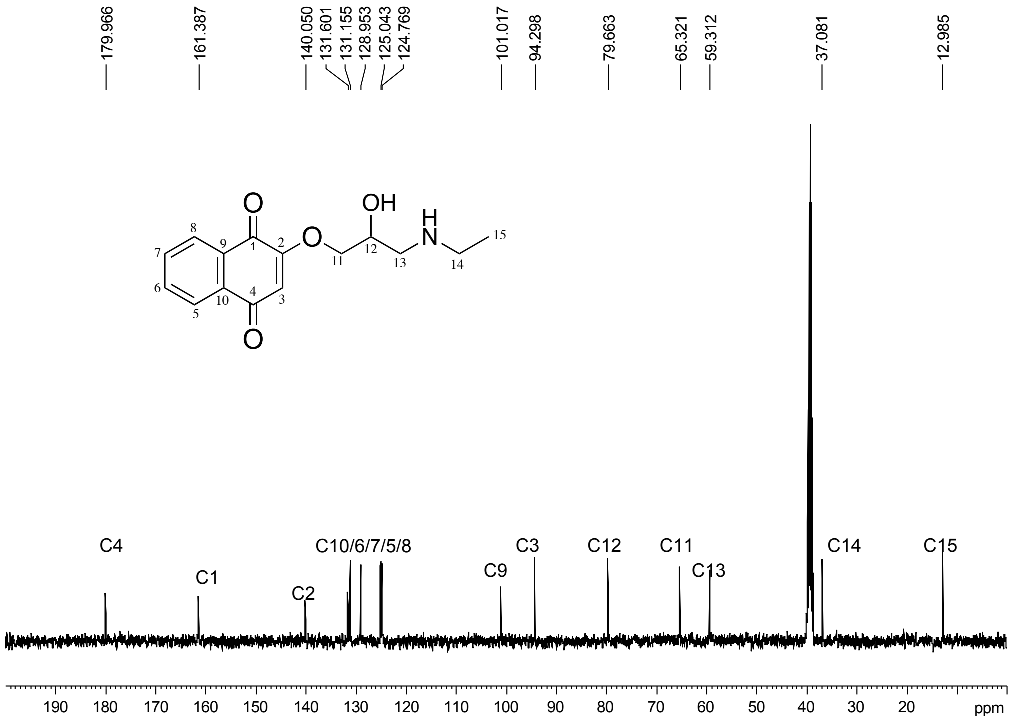


**Figure S20:** $^{13}C$ NMR spectrum (100 MHz, DMSOd6) and DEPT 135 of 2-(3-(ethylamino)-2-hydroxypropoxy)naphthalene-1,4-dione (**10a**).

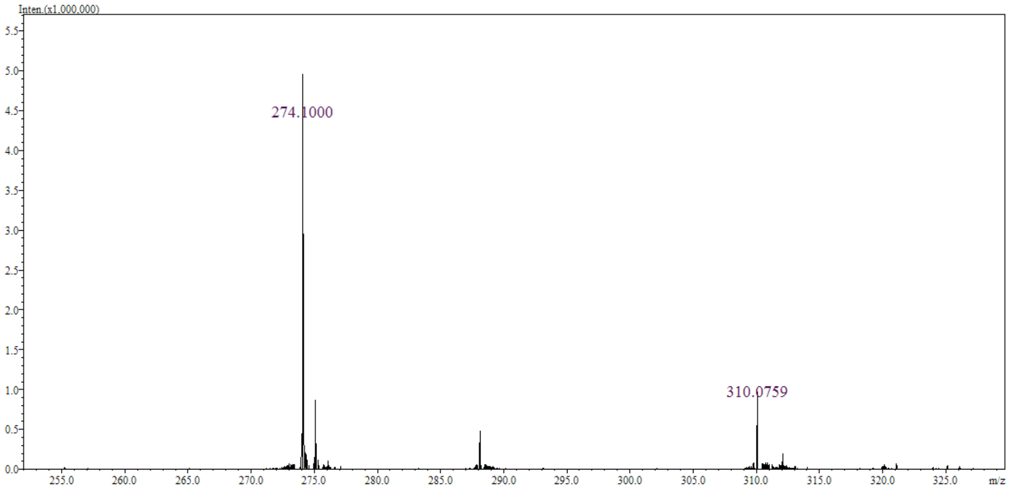


**Figure S21:** HRMS spectrum of 2-(3-(ethylamino)-2-hydroxypropoxy)naphthalene-1,4-dione (**10a**).

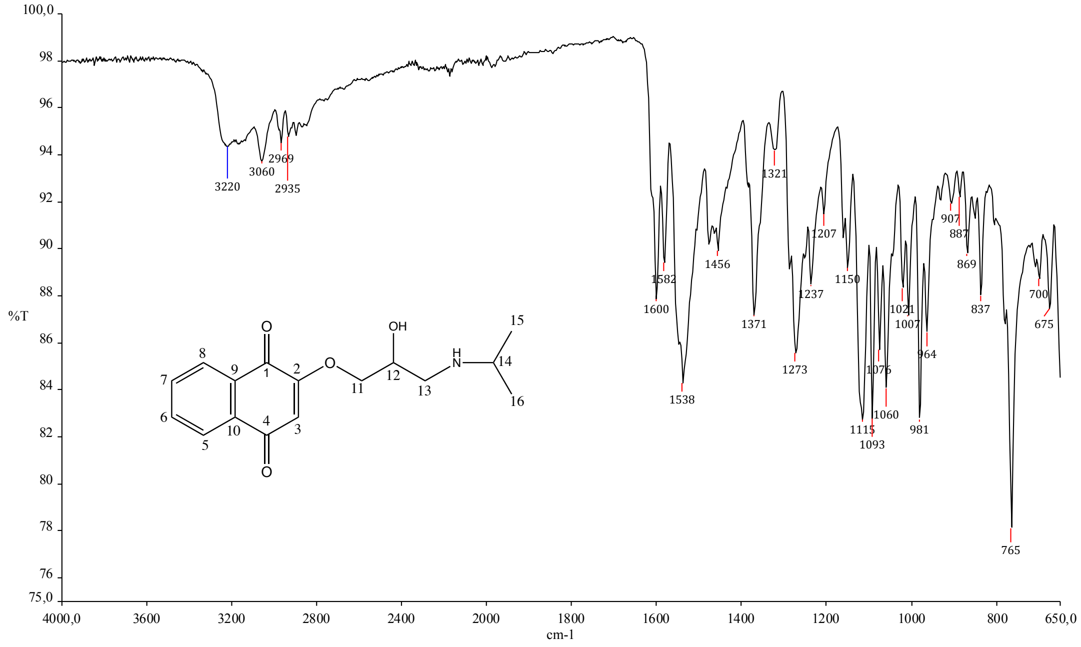

**Figure 22:** IR (ATR) spectrum of 2-(2-hydroxy-3-(isopropylamino)propoxy)naphthalene-1,4-dione (**10b**).

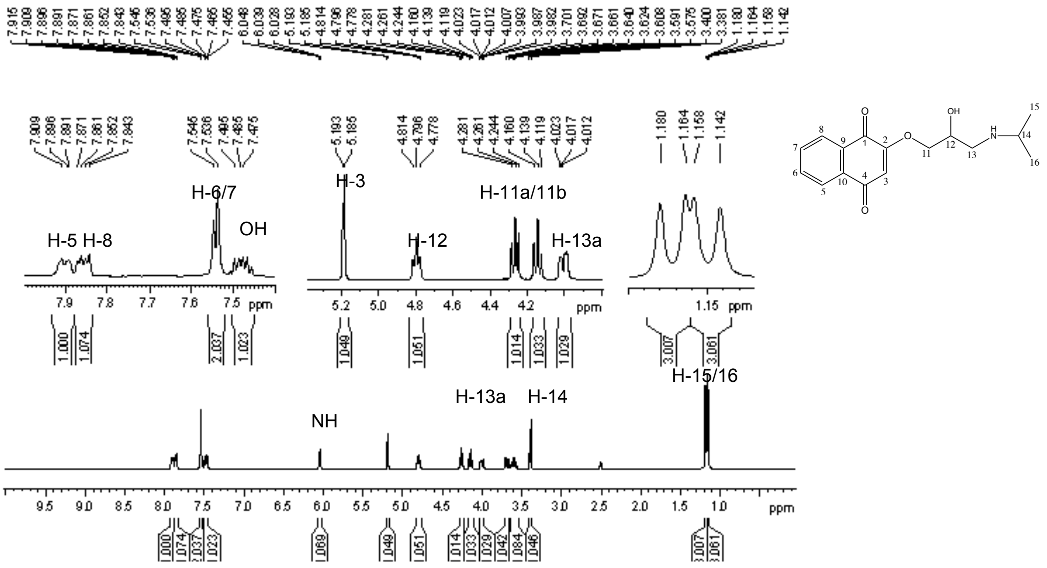

**Figure S23:** $^{1}H$ NMR spectrum (400 MHz, DMSOd6) of 2-(2-hydroxy-3-(isopropylamino)propoxy)naphthalene-1,4-dione (**10b**).

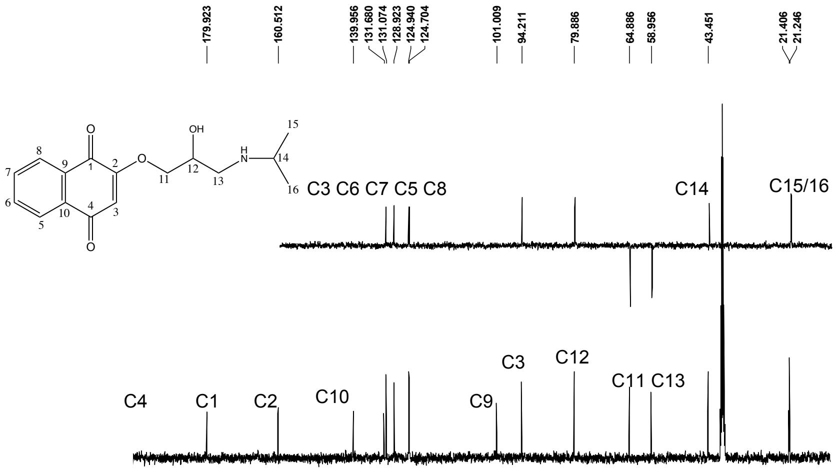


**Figure S24:** $^{13}$C NMR spectrum (100 MHz, DMSOd6) and DEPT 135 of 2-(2-hydroxy-3-(isopropylamino)propoxy)naphthalene-1,4-dione (**10b**).

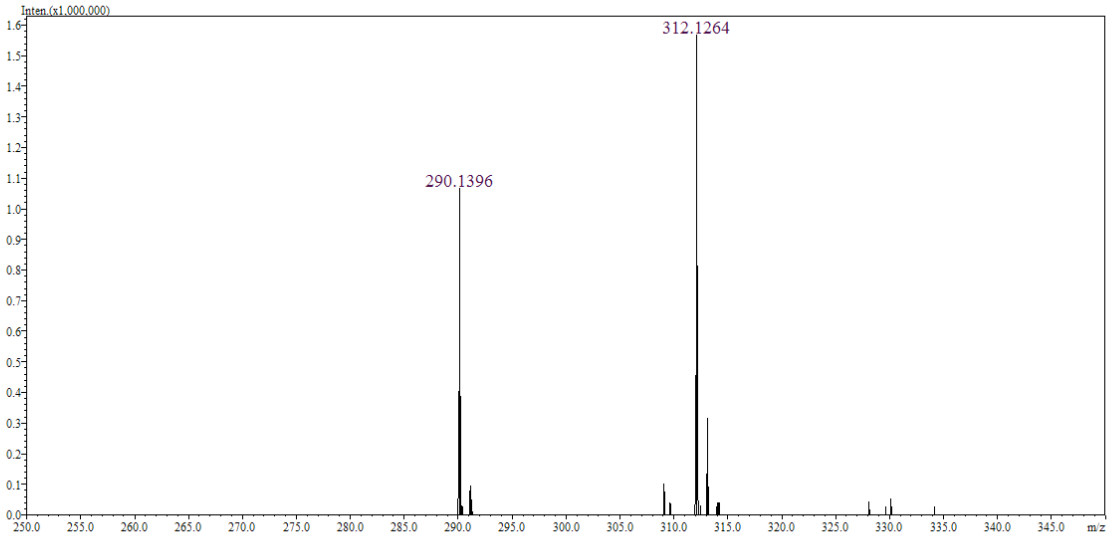


**Figure S25:** HRMS spectrum of 2-(2-hydroxy-3-(isopropylamino)propoxy)naphthalene-1,4-dione (**10b**).

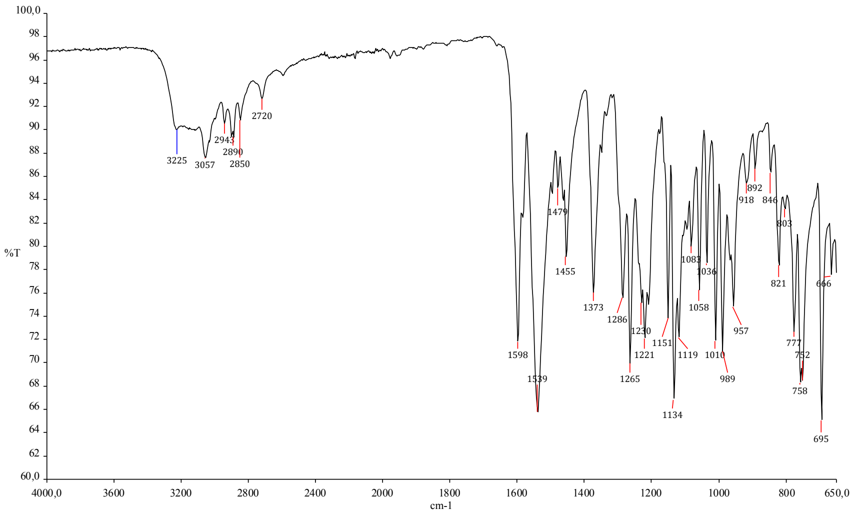

**Figure S26:** IR (ATR) spectrum of of 2-(3-(benzylamino)-2-hydroxypropoxy)naphthalene-1,4-dione (**10c**).

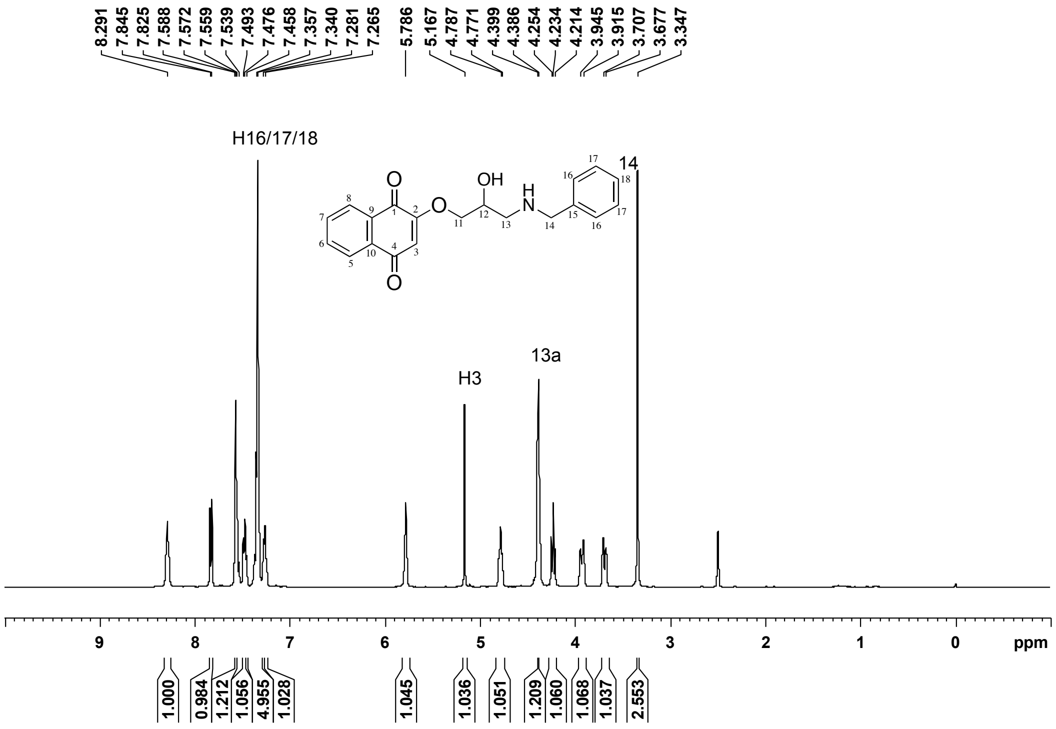

**Figure S27:** $^{1}$H NMR spectrum (400 MHz, $DMSO_{d6}$) of 2-(3-(benzylamino)-2-hydroxypropoxy)naphthalene-1,4-dione (**10c**).

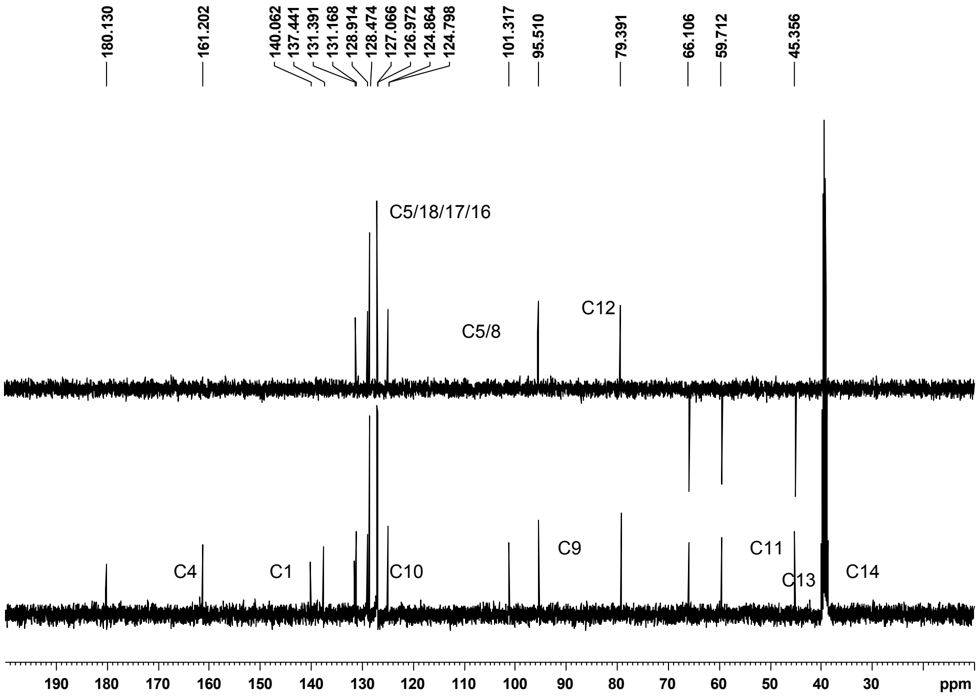


**Figure S28:** $^{13}C$ NMR spectrum (100 MHz, DMSO d6) and DEPT 135 of 2-(3-(benzylamino)-2-hydroxypropoxy)naphthalene-1,4-dione (**10c**).

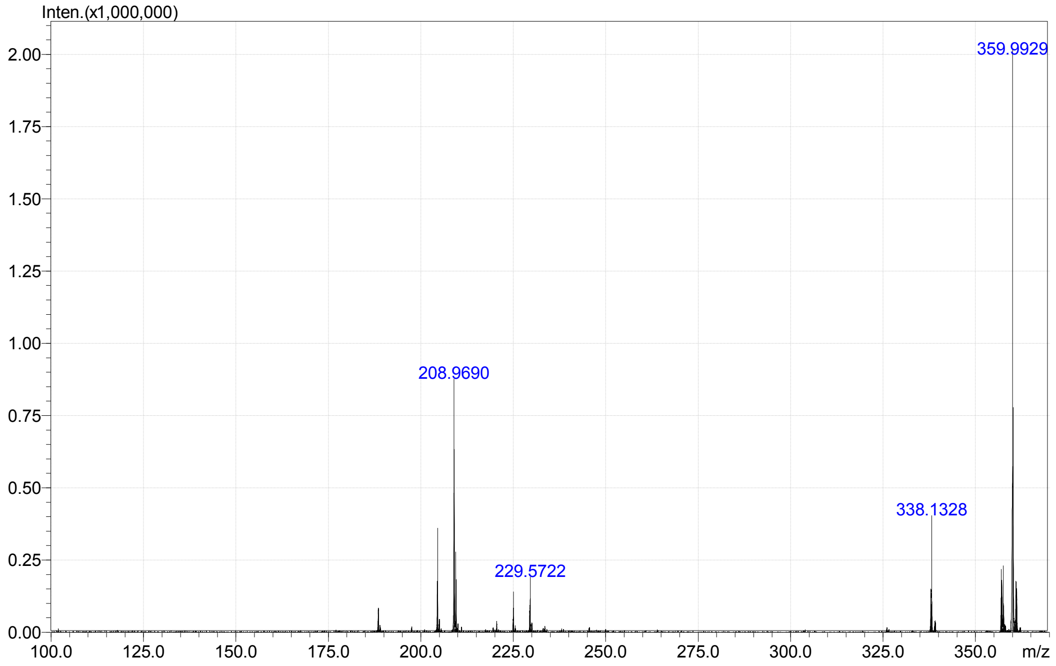


**Figure S29:** HRMS spectrum of 2-(3-(benzylamino)-2-hydroxypropoxy)naphthalene-1,4-dione (**10c**).
